# Cucumber malate decarboxylase, CsNADP-ME2, functions in the balance of carbon and amino acid metabolism in fruit

**DOI:** 10.1093/hr/uhad216

**Published:** 2023-10-25

**Authors:** Nan Shan, Youjun Zhang, Yicong Guo, Wenna Zhang, Jing Nie, Alisdair R Fernie, Xiaolei Sui

**Affiliations:** Beijing Key Laboratory of Growth and Developmental Regulation for Protected Vegetable Crops, College of Horticulture, China Agricultural University, Beijing 100193, China; Jiangxi Province Key Laboratory of Root and Tuber Crops Biology (Jiangxi Agricultural University), Nanchang 330045, China; Max Planck Institute of Molecular Plant Physiology, Potsdam-Golm 14476 Germany; Beijing Key Laboratory of Growth and Developmental Regulation for Protected Vegetable Crops, College of Horticulture, China Agricultural University, Beijing 100193, China; Beijing Key Laboratory of Growth and Developmental Regulation for Protected Vegetable Crops, College of Horticulture, China Agricultural University, Beijing 100193, China; Beijing Key Laboratory of Growth and Developmental Regulation for Protected Vegetable Crops, College of Horticulture, China Agricultural University, Beijing 100193, China; Max Planck Institute of Molecular Plant Physiology, Potsdam-Golm 14476 Germany; Beijing Key Laboratory of Growth and Developmental Regulation for Protected Vegetable Crops, College of Horticulture, China Agricultural University, Beijing 100193, China

## Abstract

Central metabolism produces carbohydrates and amino acids that are tightly correlated to plant growth and thereby crop productivity. Malate is reported to link mitochondrial respiratory metabolism with cytosolic biosynthetic pathways. Although the function of malate metabolism-related enzymes in providing carbon has been characterized in some plants, evidence for this role in the fleshy fruit of cucumber is lacking. Here, radiolabeled bicarbonate fed into the xylem stream from the cucumber roots was incorporated into amino acids, soluble sugars, and organic acids in the exocarp and vasculature of fruits. The activities of decarboxylases, especially decarboxylation from NADP-dependent malic enzyme (NADP-ME), were higher in cucumber fruit than in the leaf lamina. Histochemical localization revealed that CsNADP-ME2 was mainly located in the exocarp and vascular bundle system of fruit. Radiotracer and gas-exchange analysis indicated that overexpression of *CsNADP-ME2* could promote carbon flux into soluble sugars and starch in fruits. Further studies combined with metabolic profiling revealed that the downregulation of *CsNADP-ME2* in RNA interference (RNAi) lines caused the accumulation of its substrate, malate, in the exocarp. In addition to inhibition of glycolysis-related gene expression and reduction of the activities of the corresponding enzymes, increased amino acid synthesis and decreased sugar abundance were also observed in these lines. The opposite effect was found in *CsNADP-ME2*-overexpressing lines, suggesting that there may be a continuous bottom-up feedback regulation of glycolysis in cucumber fruits. Overall, our studies indicate that *CsNADP-ME2* may play potential roles in both central carbon reactions and amino acid metabolism in cucumber fruits.

## Introduction

Carbon and nitrogen are the most vital elements in all living things, including plants, animals, and microbes. Plant development and the production of crops are closely associated with the regulation of carbon and nitrogen metabolism [1]. Carboxylate metabolism-related enzymes are proposed to contribute to carbon and nitrogen metabolic pathways in several plant species [2, 3]. For example, increased malic enzyme flux and lipid abundance along with altered amino acid and fatty acid levels were observed in the seeds of homozygous transgenic soybean plants that expressed *Arabidopsis* malic enzyme alleles [4]. In transgenic tomato plants, the activity of mitochondrial malate dehydrogenase is suppressed, which enhances aerial growth and carbon absorption [5]. When the NAD-dependent malic enzyme (NAD-ME) is deficient in *Arabidopsis*, excess malate is significantly diverted to amino acid production at night-time [6]. A basic metabolic pathway that is irreversible in plants is catalyzed by the enzyme phospho*enol*pyruvate carboxylase (PEPC). Reduced PEPC activity in the *Arabidopsis ppc1*/*ppc2* double mutant was shown to result in repressed ammonium absorption, impaired synthesis of malate and citrate, and higher starch and sucrose buildup, proving the critical function of PEPC in controlling the balance of carbon and nitrogen metabolism [7]. Similarly, the 2-oxoglutarate/malate translocator plays two roles in the malate valve and carbon/nitrogen metabolism in *Arabidopsis*, mediating the biosynthesis of amino acid and storage proteins in pea embryo [2, 3]. These works collectively indicate that strategies to control enzymes involved in organic acid metabolism would likely be effective means of regulating plant carbon/nitrogen metabolism.

Malate is a prominent metabolite that occupies a crucial node in the regulation of the metabolism of carbon in plants. It relates mitochondrial respiratory metabolism to cytosolic biosynthetic pathways [8]. Malate has been shown to exert important functions in the tricarboxylic acid (TCA) cycle and metabolite signaling [9, 10]. The oxidative decarboxylation of malate to create pyruvate, CO_2_, and reduced nicotinamide adenine dinucleotide phosphate (NADPH) is catalyzed by the NADP-dependent malic enzyme (NADP-ME), which has also been linked to normal plant growth and stress responses [*11*–*13*]. In plants, NADP-ME is encoded by multiple genes with different expression patterns and biochemical properties, whose gene products are localized in either the cytosol or the plastid [*14*–*16*]. In *Arabidopsis*, three cytosolic informs (AtNADP-ME1–3) and a plastidic isoform (AtNADP-ME4) were identified [15]. Following phylogenetic analysis, AtNADP-ME1 clusters in the same group with maize (*Zea mays*) cytosolic NADP-ME (ZmCytNADP-ME) [17], which has similar expression patterns and kinetic properties [18]. AtNADP-ME2 shares 90% identity with AtNADP-ME3 at the protein level, and both group with cytosolic dicot sequences in a phylogenetic tree [15]. The plastidic isoform AtNADP-ME4 resembles maize plastidic isoforms (ZmC_4_-NADP-ME and Zm-nonC_4_-NADP-ME) in kinetic characterization and groups with plastidic dicot sequences in a phylogenetic tree [17], suggesting that a C_3_ plastidic isoform may be an ancestor of the photosynthetic and non-photosynthetic plastidic isoforms found in C_4_ plants [18]. Although *Arabidopsis* isoforms are highly homologous, they differ in expression trends and function [14, 15]. AtNADP-ME1 is confined to maturing seeds and secondary roots, and is necessary in the abscisic acid response and seed germination during dry storage [19, 20]. AtNADP-ME2 is responsible for the major enzymatic activity in all mature organs. This protein is implicated in sugar metabolism in veins [21], in the production of reactive oxygen species (ROS) [22], and in maintaining redox and carbon cellular balances in plants [23]. AtNADP-ME3 is exclusively expressed in trichomes and pollens [15]. AtNADP-ME4 is expressed constitutively in reproductive or vegetative organs and plays a critical role in lipid metabolism [14, 15].

In plants, NADP-ME is reported to be regulated by several processes, according to extensive research. Some plants possess regulatory components that affect the expression of the *NADP-ME* gene in a cell-specific manner [24]. For example, in transgenic tobacco plants it has been discovered that the promoter of the common bean (*Phaseolus vulgaris*) *NADP-ME* gene strongly regulates expression in tissues such as those surrounding the vascular, floral, and reproductive systems [25]. Another example is the promoters of *AtNADP-ME2* and *AtNADP-ME4* from *Arabidopsis*, which were verified to direct pronounced expression to the middle vein and, to variable degrees, to the minor veins [21]. Likewise, an ancestral G-box recognized by basic helix–loop–helix transcription factors in the *NADP-ME* promoter of the C_4_ crop maize is also present in C_3_ species [26]. RPM1-INDUCED PROTEIN KINASE (RIPK), a receptor-like cytoplasmic kinase, may directly phosphorylate and activate AtNADP-ME2 to sustain ROS production in *Arabidopsis* [27].

The rapid development and growth of fleshy cucumber (*Cucumis sativus*) fruit require significant sources of carbon and nitrogen. Researchers have reported that monosaccharides like glucose and fructose are the primary carbohydrates that accumulate in mature cucumber fruit [28, 29]. We previously reported the coincidentally rapid accumulation of organic acids and increased PEPC activity during cucumber fruit development [30]. Our results also indicated that cucumber fruits displayed complicated vascular anatomy [31] and complex photosynthetic characteristics [30]. Peripheral (PeVB), main (MVB), carpel (CVB), and placental vascular bundles (PlVB) are among the four sets of functionally differentiated vascular bundles from the outside to the inside of the fruits of cucumber plants, which are distinguished from the vascular networks in the leaves, petiole, stems, and peduncle [31, 32]. However, so far, the *in vivo* functions of CsNADP-ME in the metabolism of nitrogen and carbon in cucumber fruits are not clear. Here, we used a combination of transgenesis, ^14^C feeding, and cellular localization experiments as well as metabolic profiling to illustrate the pivotal role of the malate decarboxylase-encoding gene *CsNADP-ME2* in carbon reaction and amino acid metabolism of cucumber fruit. Taken together, our study reveals a potential mechanism by which malate decarboxylase may act in the network integration of carbohydrate synthesis and primary metabolism in this non-foliar organ. Our study also offers a promising approach for enhancing the yield and quality of fleshy fruit in cucumber.

## Results

### Cucumber fruit contained considerable proportions of radiolabel generated from root-fed ^14^C-bicarbonate

**Figure 1:**
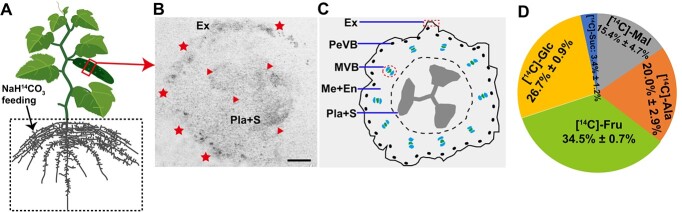
Autoradiography and proportion of radiolabel materials in cucumber fruits. **A** Cucumber root was fed with 3.7 × 10^6^ Bq NaH^14^CO_3_. A mature plant with fruit was treated. **B**^14^C autoradiograph of fruit after [^14^C]NaHCO_3_ was supplied to the xylem stream. Stars and triangles indicate autoradiography signals. Scale bar: 0.5 cm. **C** Diagram of fruit cross-section of (**B**). Total incorporated material in cucumber fruit was determined, and TLC was used to isolate soluble metabolites. Scintillation counting was subsequently used to measure the percentage of radiolabel in major spots. **D** Percentages of all radionuclides found in a metabolite are represented by the data, which are presented as means and standard errors (*n* = 3). Ala, alanine; En, endocarp; Ex, exocarp; Fru, fructose; Glc, glucose; Mal, malate; Me, mesocarp; MVB, main vascular bundle; PeVB, peripheral vascular bundle; Pla, placenta; S, seed; Suc, sucrose.

According to several studies, organic acids from the transpiration stream may supply carbon to cells associated with the veins of plants including rice, tobacco, and *Arabidopsis*, and those cells with high C_4_ acid decarboxylase activity may rapidly release CO_2_ [21, 24, 33, 34]. Cucumber fruits contain four sets of vascular bundles with intricate structural connections [31]. Here, we speculate whether these types of vascular cells in cucumber fruits could play a synergistic role in the reuse of carbon from substrates supplied to the stream of transpiration. To test this, first we supplied ^14^C-labelled bicarbonate ([^14^C]NaHCO_3_) to the xylem stream via the root of cucumber plants (Fig. 1A). The radionuclides were accordingly detected in the exocarp, PeVB, MVB, and placenta tissues of cucumber fruits (Fig. 1B and C). Secondly, thin-layer chromatography (TLC) followed by scintillation counting was employed to isolate and quantify radiolabel in soluble substances to further determine the fate of ^14^C. High concentrations of radiolabel derived from [^14^C]NaHCO_3_ were identified in amino acids such as alanine (~20.0% of total soluble material) and organic acids such as malate (~15.4% of total soluble material). However, the majority was present as soluble sugars, particularly hexoses (Fig. 1D). Specifically, fructose represented ~34.5% of the total soluble material, followed by glucose (~26.7%) and sucrose (~3.4%) (Fig. 1D). It seems likely that the cells surrounding the vascular tissues of cucumber fruits could be supplied with carbon from C_4_ acid via xylem transpiration.

### Spatiotemporal expression and enzyme activity of decarboxylases in cucumber fruits

In cucumber, our data from laser capture microdissection-derived RNA sequencing (RNA-seq) revealed that the genes encoding C_4_ acid decarboxylases, such as phospho*enol*pyruvate carboxykinase (PEPCK), NAD-dependent malic enzyme (NAD-ME), and NADP-ME [31, 32], were exclusively expressed in the vasculature in the stem, petiole, and fruit (Supplementary Data Fig. S1), and the mRNA level of *CsNADP-ME2* was significantly higher than that of other C_4_ acid decarboxylase genes (Supplementary Data Fig. S1). Four candidate *CsNADP-ME* genes were identified in cucumber (Supplementary Data Fig. S2A) and named according to their homologs in *Arabidopsis* [15]. Multiple sequence alignment of *Arabidopsis* AtNADP-ME1–4 with cucumber orthologs indicated 76.60% homology (Supplementary Data Fig. S2A), and showed strong conservation of residues involved in the active site binding of malate or pyruvate (Supplementary Data Fig. S2A) [4, 35]. NADP-MEs in plants can be classified into four groups [15]: groups I and II comprise cytosolic and plastidic isoforms from dicots, respectively; group III contains isoforms from monocots; and group IV is composed of isoforms from both monocots and dicots, e.g. CsNADP-ME2 and AtNADP-ME1 (Supplementary Data Fig. S2B). Phylogenetic analysis revealed high similarity between cucumber and *Arabidopsis* orthologs (Supplementary Data Fig. S2B).

**Figure 2:**
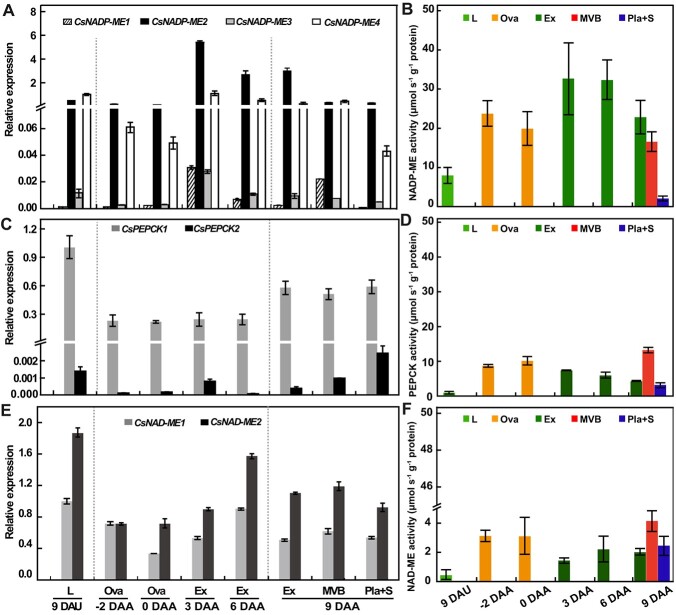
Gene expression profiles of decarboxylases and enzyme activities in cucumber fruit. Transcript levels (**A**, **C**, **E**) and enzymatic activities (**B**, **D**, **F**) derived from *CsNADP-ME* (**A**, **B**), *CsPEPCK* (**C**, **D**), and *CsNAD-ME* (**E**, **F**) were analyzed. RT–qPCR was conducted on cDNA derived from mature cucumber leaves and different fruit tissues. Specific primers employed for each gene family member are displayed in Supplementary Data Table S2, and primers for *β-tubulin* were used in a duplex PCR reaction. Enzymatic activities were calculated per protein concentration. Error bars denote standard deviation, *n* = 3. DAA, days after anthesis; DAU, days after unfolding (of leaves); Ex, exocarp; L, leaf; MVB, main vascular bundle; Ova, ovary; Pla, placenta; S, seed.

We next investigated gene expression and enzyme activity of these three decarboxylases (CsNADP-ME, Fig. 2A and B; CsPEPCK, Fig. 2C and D; and CsNAD-ME, Fig. 2E and F) in cucumber fruits and leaf lamina as control. Among the *CsNADP-ME* genes, both *CsNADP-ME2* and *CsNADP-ME4* were expressed at high levels, while *CsNADP-ME1* and *CsNADP-ME3* were barely detectable in leaves and fruits (Fig. 2A), which is consistent with the RNA-seq data (Supplementary Data Fig. S1). Beyond this, *CsNADP-ME2* transcripts (Fig. 2A) and total NADP-ME activity (Fig. 2B) displayed higher levels in cucumber fruits. In fruits at 9 days after anthesis (DAA), both transcript levels and enzymatic activity of NADP-ME were higher in exocarp tissue, followed by MVB and placenta tissues (Fig. 2A and B). In addition, fruits expressed *CsPEPCK1* at a lower level than leaves, but *CsPEPCK2* transcripts were barely present in either tissue (Fig. 2C). In contrast to the transcriptional pattern, the PEPCK enzyme activity was 4- to 12-fold higher in fruits than leaves, with MVB tissue from 9-DAA fruits exhibiting the highest activity level (Fig. 2D). Transcripts of *CsNAD-ME* family genes were similar in leaves and fruits of different developmental stages (Fig. 2E); however, NAD-ME activity was 2- to 7-fold higher in fruits than in leaves, with the highest level again recorded in MVB tissue (Fig. 2F). Similar to the changes in transcript expression patterns and enzymatic activities of the three decarboxylases, *CsNADP-MDH* (*NADP-dependent malate dehydrogenase*) (Supplementary Data Fig. S3A and B) and the previously reported *PPC* family gene encoding PEPC [phosphoenolpyruvate (PEP) carboxylase] [30], as well as *CsPPDK* (*pyruvate orthophosphate dikinase*) (Supplementary Data Fig. S3C and D), showed lower transcripts but higher enzymatic activities in fruits when compared with leaf lamina. Overall, in cucumber fruits, *CsNADP-ME2* was the most highly expressed member of the genes encoding the C_4_ acid decarboxylases (Fig. 2A–F; Supplementary Data Fig. S1). Therefore, we next focused on the potential function of the *CsNADP-ME2* gene in cucumber fruits.

### *CsNADP-ME2* is highly expressed in the exocarp, vasculature, and placenta of cucumber fruit

**Figure 3:**
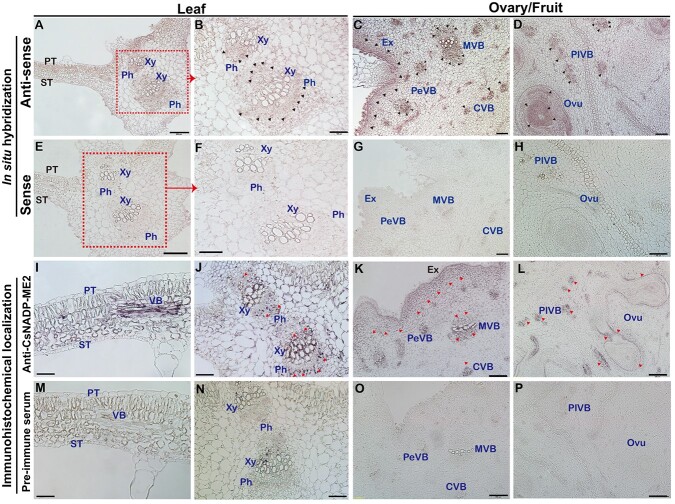
Localization of *CsNADP-ME2* gene and CsNADP-ME2 protein in cucumber leaves and fruits. **A**–**H***In situ* hybridization detection of *CsNADP-ME2* transcripts. Cross-sections of cucumber leaves (**A**, **B**, **E**, **F**) and ovaries/fruits (**C**, **D**, **G**, **H**) hybridized with *CsNADP-ME2* antisense (**A**–**D**) and sense (**E**–**H**) probes, respectively. (**B**) and (**F**) are the enlargements of (**A**) and (**E**), as shown by the dotted frame. Triangles in (B), (C), and (D) indicate positive signals. **I**–**P** Immunohistochemical localization of CsNADP-ME2. Cross-sections of cucumber leaves (**I**, **J**) and ovaries/fruits (**K**, **L**) incubated with anti-CsNADP-ME2 antibody and subsequent alkaline phosphatase (AP)-coupled secondary antibody. Pre-immune serum was used to incubate cross-sections as the control (**M**–**P**). Triangles in (J), (K), and (L) highlight the signals. Sections were prepared from leaves 1 day after unfolding (DAU) and ovaries/fruits from −2 DAA to 0 DAA. Scale bars: 50 μm in (**I**, **M**), 100 μm in (**B**–**D**, **F**–**H**, **J**, **N**), 200 μm in (**A**, **E**, **K**, **L**, **O**, **P**). Ex, exocarp; Ovu, ovule; Xy, xylem; ST, spongy tissue, PT, palisade tissue; Ph, phloem; VB, vascular bundle; PeVB, MVB, CVB and PlVB, peripheral, main, carpel and placental vascular bundle.

Considering the enhanced expression of *CsNADP-ME2* in the exocarp, vasculature, and placenta tissues of cucumber fruit (Fig. 2A), the spatial and tissue-specific localization of *CsNADP-ME2* and its protein in cucumber leaf and ovary/fruit was further examined using *in situ* hybridization and immunohistochemical localization methods, respectively (Fig. 3). *In situ* hybridization results indicated that *CsNADP-ME2* transcripts were mainly located in the cells within and/or around the vein and palisade tissues of leaves (Fig. 3A, B, E, and F), as well as in the exocarp, ovule, and four sets of vascular bundle systems of fruits, namely PeVB, MVB, CVB and PlVB (Fig. 3C, D, G, and H). Western blotting was conducted to assess the antisera’s potency, and the findings revealed that the anti-*CsNADP-ME2* antiserum was specifically bound to *CsNADP-ME2* (Supplementary Data Fig. S4). Localization patterns of the CsNADP-ME2 protein, by using a secondary antibody conjugated to either alkaline phosphatase (AP) (Fig. 3I–P) or fluorescein isothiocyanate (FITC) (Supplementary Data Fig. S5), matched those in their respective transcripts (Fig. 3A–H).

### Manipulating expression of *CsNADP-ME2* influences metabolic fluxes and respiratory gas exchange in transgenic cucumber fruits

**Figure 4:**
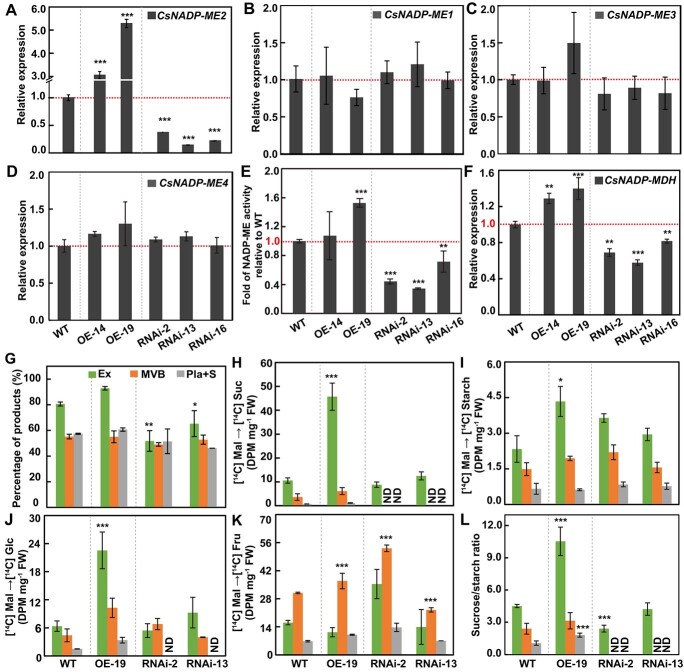
Manipulating the expression of *CsNADP-ME2* regulates carbon flux in transgenic cucumber fruits. **A**–**F** Expression analysis of *CsNADP-ME2* (**A**), *CsNADP-ME1* (**B**), *CsNADP-ME3* (**C**), *CsNADP-ME4* (**D**), and *CsNADP-MDH* (**F**) and enzymatic activity of NADP-ME (**E**) in exocarp tissues from WT plants, RNA interference lines (RNAi-2, RNAi-13, and RNAi-16), and overexpression lines (OE-14 and OE-19). Transcript levels of *CsNADP-MEs* (**A**–**D**) and *CsNADP-MDH* (**F**) were measured by using *β-tubulin* as the internal reference. (**G**–**L**) Metabolic flux analysis in fruits of WT and *CsNADP-ME2* transgenic lines. Three transgenic lines (OE-19, RNAi-2, and RNAi-13) were chosen for the study while WT plants served as the control. Tissues of exocarp (Ex), main vascular bundle (MVB), and placenta and seeds (Pla + S) from WT and transgenic lines were fed [U^14^C] malate. **G** Percentage of [U^14^C] malate-derived material. **H**–**K** Incorporation of [U^14^C] malate into sucrose (**H**), starch (**I**), glucose (**J**), and fructose (**K**). **L** Sucrose/starch ratio. According to Tukey’s test, statistically significant differences from WT are indicated by the means (*n* = 3) followed by asterisks (*n* = 3) (^*^P < .05, ^**^P < .01, ^***^P < .001). Fru, fructose; Glc, glucose; Mal, malate; NADP-MDH, NADP-dependent malate dehydrogenase; ND, not detected; Suc, sucrose.

To further analyze the role of *CsNADP-ME2* in cucumber fruits, overexpression (OE) and RNA interference (RNAi) constructs of *CsNADP-ME2* (Supplementary Data Fig. S6A and B) were generated and subsequently independently transformed into cucumber cotyledon. Four OE lines and five RNAi lines were selected from more than 20 independent *T*_0_ transgenic plants (Supplementary Data Fig. S6C). Then, two independent OE *T*_2_ lines (OE-14 and OE-19) and three RNAi *T*_2_ lines (RNAi-2, RNAi-13, and RNAi16) were selected based on their *CsNADP-ME2* expression levels for further study. Real-time quantitative PCR (RT–qPCR) analysis illustrated that ~3-, 5-, 0.30-, 0.15-, and 0.20-fold transcript levels were detected in OE-14, OE-19, RNAi-2, RNAi-13, and RNAi-16 lines, respectively, in contrast to wild-type (WT) plants (Fig. 4A), whereas the expression of the other three members comprising *CsNADP-ME1*, *3*, and *4* barely changed (Fig. 4B–D). Meanwhile, the total NADP-ME activity obviously increased in line OE-19 but significantly decreased in all three RNAi lines when compared with WT (Fig. 4E), suggesting that *CsNADP-ME2* is most likely the main gene responsible for NADP-ME activity in the oxidative decarboxylation of malate. In addition, when compared with WT, the OE lines had higher expression levels of *CsNADP-MDH* while the RNAi-2 and RNAi-13 lines had lower expression levels (Fig. 4F). Taking these results together, the expression of genes involved in the metabolism of malate may be significantly impacted by alterations in *CsNADP-ME2*. Based on the transcripts of *CsNADP-ME2* and other related genes, as well as total *NADP-ME* activity, we selected lines OE-19, RNAi-2, and RNAi-13 for further analysis.

We next assessed the metabolic fluxes of carbohydrates by incubating excised pericarp discs, MVB, and placenta and seed tissues from nine DAA fruits in a buffered medium containing 10 mM [U^14^C] malate. After incubation, the discs were rinsed and frozen before the labeled material was fractionated to ascertain the label redistribution. The percentage of [U^14^C] malate derivatives was higher in the OE line, but lower in the RNAi lines (Fig. 4G). Specifically, [^14^C] sucrose (Fig. 4H), [^14^C] starch (Fig. 4I), and [^14^C] glucose (Fig. 4J) were significantly accumulated in the exocarp tissues of the OE line compared with WT, while [^14^C] fructose abundance rose in MVB of the OE line and one out of two RNAi lines (Fig. 4K). Moreover, overexpression of *CsNADP-ME2* significantly increased the sucrose-to-starch ratio in cucumber fruits compared with the WT, whereas in *CsNADP-ME2*-RNAi lines obvious decreases were observed in the sucrose/starch ratio (Fig. 4L), indicating that the changes of *CsNADP-ME2* gene expression (Fig. 4A) and enzyme activity (Fig. 4E) had larger effects on sucrose levels than on the starch content in the transgenic cucumber plants. In general, these results suggest that manipulating the expression of *CsNADP-ME2* may influence carbon metabolism by regulating carbon flux into starch and soluble sugars.

**Figure 5:**
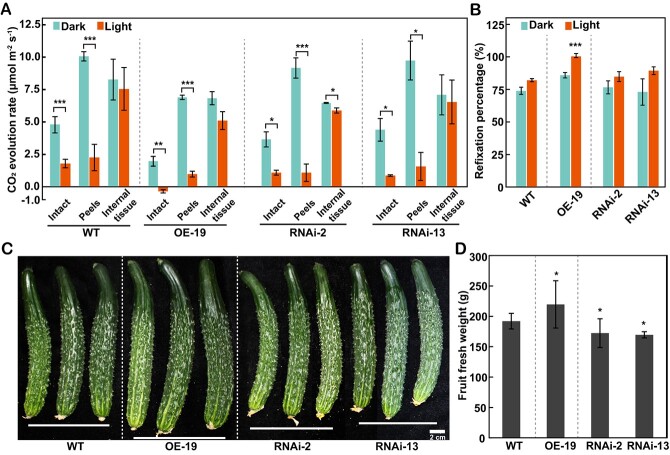
Carbon reactions of photosynthesis in *CsNADP-ME2*-transgenic cucumber fruits. Three transgenic lines (OE-19, RNAi-2, and RNAi-13) were included in the study, whereas WT plants served as the control. **A** CO_2_ evolution rates of entire fruit, peels, and interior tissue in dark and light environments (300 μmol quanta m^−2^ s^−1^ irradiance). The device automatically regulated the ambient CO_2_ content and air temperature at 400 ± 10 μmol mol^−1^ and 27 ± 1°C. Data were computed on the basis of fruit surface area. **B** Calculated CO_2_ refixation percentage in fruits of WT and *CsNADP-ME2*-transgenic lines. **C**, **D** Phenotypic analysis of fruits from WT and *CsNADP-ME2* transgenic plants. Fruits were digitally extracted for comparison (**C**). Scale bar: 2 cm. **D** Fruit fresh weight of WT and *CsNADP-ME2*-transgenic plants. Values represent the average of eight biological replicates. Based on Tukey’s test, the means (*n* = 3) followed by asterisks show significant variations (^*^P < .05, ^**^P < .01, ^***^P < .001).

Given the change of transcript and metabolite levels, gas exchange analysis in the fruits of transgenic cucumber was conducted according to Sui *et al*. [30]. These results revealed that a large amount of CO_2_ was released from peels, internal tissues, and intact fruits in both WT plants and transgenic lines in the dark condition (Fig. 5A). By contrast, in the light, although net photosynthesis could not be detected in fruit tissues, CO_2_ evolution decreased significantly compared with that in the dark, especially in the intact fruits and peel tissues rich in photosynthetic pigments, suggesting the occurrence of CO_2_ absorption by the fruits under the light condition (Fig. 5A). Furthermore, re-fixation of respiratory CO_2_ by the fruits was analyzed in the light and dark conditions according to methods described in a previous report [30], and was found to be significantly improved in the OE line in the light condition when compared with the WT (Fig. 5B). Accordingly, in contrast to WT cucumber, fruit weight rose by 2–16% in the OE line and dropped by 2–10% in the RNAi lines (Fig. 5C and D), indicating the potential contribution of *CsNADP-ME2* to the carbon acquisition of the fruit.

### Altered *CsNADP-ME2* mediates amino acid metabolism in cucumber fruits

**Figure 6:**
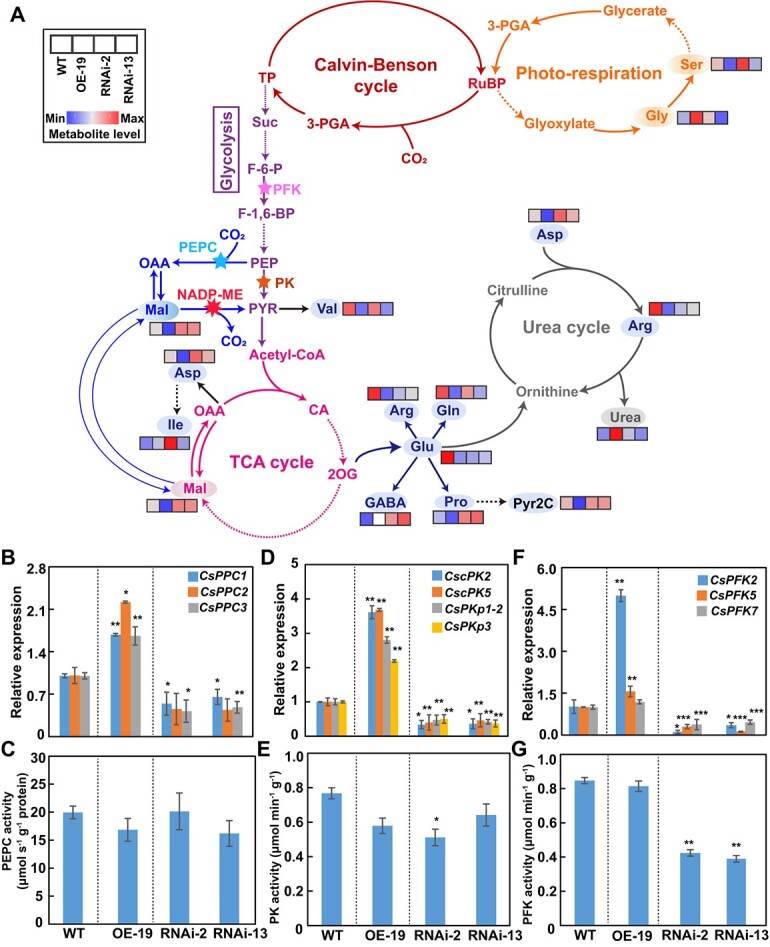
Analysis of altered primary metabolites and gene expression in *CsNADP-ME2* transgenic cucumber fruits. Three transgenic lines—OE-19, RNAi-2, and RNAi-13—were included in the investigation, with WT plants serving as the control. **A** Putative biological pathways related to metabolism of nitrogen and carbon in cucumber fruit. The metabolic pathways were drawn according to the KEGG Pathway Database (https://www.kegg.jp/kegg/pathway.html). The metabolic reactions shown are involved in the Calvin–Benson cycle, photorespiration, the TCA cycle, glycolysis, and the urea cycle. Solid arrows indicate metabolic flux. Dotted arrows indicate that the process consists of multiple catalytic reactions. Stars represent the catalytic reactions of NADP-ME, PEPC, PFK, and PK, respectively. Metabolite levels are indicated in the small squares by colors ranging from blue to red, from low to high. Data on metabolites are normalized to mean levels for WT. Results are means of five replications. **B**–**G** Transcript levels (**B**, **D**, **F**) and enzymatic activities (**C**, **E**, **G**) of *CsPPC* (**B**, **C**), *CsPK* (**D**, **E**), and *CsPFK* (**F**, **G**) in exocarp tissue from WT and *CsNADP-ME2*-transgenic lines. RT–qPCR of each gene family member was amplified by specific primers displayed in Supplementary Data Table S2. The enzymatic activity of PEPC was calculated per protein concentration, and the enzymatic activities of PF and PFK were calculated per fresh weight. Means (*n* = 3) followed by asterisks in (**B**)–(**G**) show variations from WT that are statistically significant according to Tukey’s test (^*^P < .05, ^**^P < .01, ^***^P < .001). Acetyl-CoA, acetyl coenzyme A; Arg, arginine; Asp, aspartate; CA, citric acid; FA, fumaric acid; Fru, fructose; F-1,6-BP, fructose-1,6-bisphosphate; F-6-P, fructose-6-phosphate; GABA, γ-aminobutyric acid; Glc, glucose; Gln, glutamine; Glu, glutamate; Gly, glycine; IA, isocitric acid; Ile, isoleucine; 2OG, 2-oxoglutarate; PEP, phosphoenolpyruvate; PPC/PEPC, phosphoenolpyruvate carboxylase; PFK, 2-PGA, 2-phosphoglyceric acid; 3-PGA, 3-phosphoglyceric acid; PK, pyruvate kinase; Pro, proline; PYR, pyruvic acid; ATP-dependent phosphofructokinase; Pyr2C, 1-pyrroline-2-carboxylate; RuBP, ribulose-1,5-bisphosphate; OAA, oxaloacetate; SA, succinic acid; Ser, serine; G-6-P, glucose 6-phosphate; Suc, sucrose; Mal, malate; TP, triose phosphate, an equilibrium mixture of glyceraldehyde 3-phosphate and dihydroxyacetone phosphate in carbohydrate metabolism; Val, valine.

To further comprehend the roles played by *CsNADP-ME2*, we additionally determined the intermediate metabolite content in the exocarp (Fig. 6A), MVB, and placenta tissues (Supplementary Data Fig. S7) from *CsNADP-ME2* transgenic lines and WT. In plants, the carbon skeleton required for amino acid biosynthesis can be produced through the TCA cycle and glycolysis (Fig. 6A). It has been demonstrated that the level of fructose-6-phosphate (F-6-P) phosphorylation [catalyzed by 6-phosphofructokinase (PFK)] and PEP turnover regulate *in vivo* glycolysis [36]. PEP can be converted into either pyruvate or malate catalyzed by pyruvate kinase (PK) or PEPC (and subsequent action of NADP-MDH), respectively. Malate and pyruvate may both enter the TCA cycle, hence these intermediates and their products, including malate, citrate, 2-oxoglutarate, and glutamate, feedback-limit the enzymatic activities of PK and PEPC. Therefore, in plants, the control of glycolysis comes from the bottom up, with primary regulation at the level of PEP metabolism by PK and PEPC and secondary regulation being exerted by PEP at the conversion of F-6-P to fructose-1,6-bisphosphate (F-1,6-BP) catalyzed by the ATP-dependent PFK [37].

Based on the above considerations, we next analyzed PEPC, PK, and PFK enzyme activity and gene expression in exocarp tissue from WT and *CsNADP-ME2* transgenic lines (Fig. 6B–G). In RNAi plants characterized by the downregulation of *CsNADP-ME2* expression and enzyme activity (Fig. 4A and E), the substrate malate accumulated significantly (Fig. 6A). Accumulated malate could feedback-inhibit the gene expressions and activities of PEPC and PK (Fig. 6B–E), resulting in excess of substrate PEP. The gene expression and activity of PFK (Fig. 6F and G) were subsequently feedback-inhibited by accumulated PEP, which eventually suppressed glycolysis. By contrast, due to the decrease in malate content in *CsNADP-ME2*-OE lines (Fig. 6A), the increased transcript levels and maintained enzyme activities of PEPC, PK, and PFK (Fig. 6B–G) resulted in a continuous bottom-up feedback promotion of glycolysis. These results indicated that *CsNADP-ME2* most likely mediates the feedback regulation of the glycolytic pathway in cucumber fruit.

**Figure 7:**
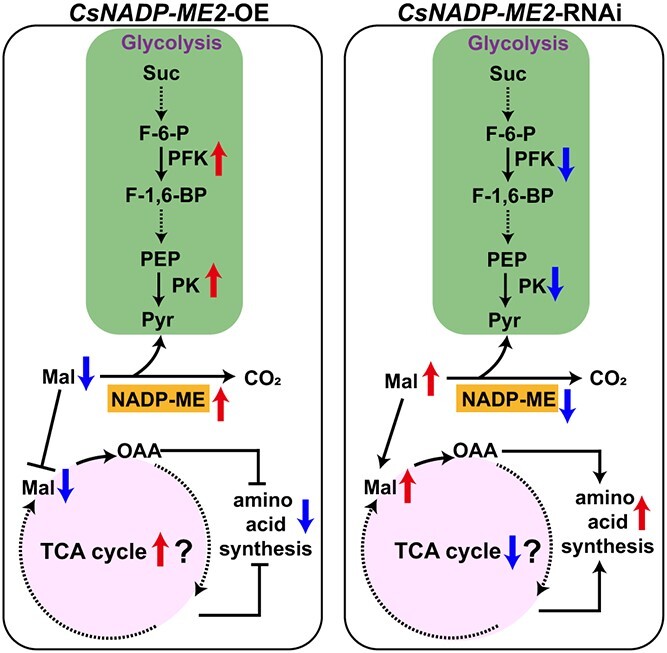
Putative model of *CsNADP-ME2* function in cucumber fruit. Based on gene expression, tissue-specific localization, activity assay of enzymes, and related physiological speculations, the role and active sites of NADP-ME (one of the decarboxylases), the TCA cycle and glycolysis in cucumber fruit are marked. Solid black arrows indicate metabolic flux. Dotted black arrows indicate that the process consists of multiple catalytic reactions. In *CsNADP-ME2*-OE plants (left) with upregulation of *CsNADP-ME2* expression and enzyme activity (up arrow) when compared with WT plants, the content of substrate malate decreased (down arrows), then the increased transcript levels and maintained enzyme activities of PK (the primary regulation) and PFK (the secondary regulation) (up arrows) could result in a continuous bottom-up feedback-promotion of glycolysis. On the other hand, as the more intermediate products of the TCA cycle were likely used to produce energy by its own reaction, the availability of carbon skeleton for amino acid metabolism was suppressed. Compared with wild-type plants, in RNAi plants (right) characterized by the downregulation of *CsNADP-ME2* expression and enzyme activity (down arrow), the substrate malate accumulated significantly (up arrows). Accumulated malate could feedback-inhibit the expressions and activities of PK and PFK (down arrows). In addition, downregulation of *CsNADP-ME2* in the fruit of RNAi lines might slow down the rate of TCA cycle activity due to the accumulation of the substrate malate. Some intermediates in the TCA cycle can be more effectively used as a precursor for the production of other derivatives/amino acids, thus promoting nitrogen assimilation, such as amino acid synthesis, in the exocarp. F-1,6-BP, fructose-1,6-bisphosphate; F-6-P, fructose-6-phosphate; 2OG, 2-oxoglutarate; Mal, malate; OAA, oxaloacetate; PFK, ATP-dependent phosphofructokinase; PK, pyruvate kinase; PYR, pyruvic acid; Suc, sucrose.

In addition, the downregulation of *CsNADP-ME2* in the fruit of RNAi lines might slow down the rate of TCA cycle activity as a result of the buildup of the TCA cycle substrate malate (Figs 6A and7). Some intermediates in the TCA cycle, such as oxaloacetate (OAA) and 2-oxoglutarate (2OG), can be more effectively employed as a precursor for the production of other derivatives/amino acids, such as aspartic acid (Asp), γ-aminobutyric acid (GABA) and proline (Pro) (Fig. 6A), thus promoting nitrogen assimilation in the exocarp. However, in *CsNADP-ME2*-OE plants, the intermediate products of the TCA cycle were probably used mainly to produce energy and to maintain equilibrium reaction, so the availability of carbon skeleton for amino acid metabolism was limited in *CsNADP-ME2*-OE lines (Fig. 7). Indeed, Asp, Pro, glutamine (Gln), glutamic acid (Glu), and arginine (Arg) were among the (derived) amino acids whose synthesis was remarkably reduced in the *CsNADP-ME2*-OE lines as contrasted with WT (Fig. 6A). Overall, these results illustrate that manipulating the expression of *CsNADP-ME2* would most likely result in the metabolic integration of the glycolytic pathway with the TCA cycle as well as amino acid synthesis within the complex network of primary metabolism in cucumber fruit (Figs 6 and7).

## Discussion

### Cucumber fruits accumulate high activities of decarboxylases

Cells surrounding the xylem of dicots like tobacco, celery, and *Arabidopsis* [21, 24], as well as woody species [38] and monocots such as rice [34], may take up and fix inorganic carbon surrounding roots. For example, organic acids in the transpiration stream might serve as a source of carbon, and release CO_2_ via highly active decarboxylase [21, 24, 34, 38]. In the present study, considerable redistribution of radioactive carbon was detected in sugars, amino acids, and organic acids in both green exocarp and placenta tissues of cucumber fruits following the supply of ^14^C-sodium bicarbonate to the root (Fig. 1A). Consistent with these results, data on laser capture microdissection-derived RNA-seq and RT–qPCR combined with enzyme assays revealed that transcripts and activities of decarboxylases, including NADP-ME, NAD-ME, and PEPCK, were also present in the vasculature of the petiole, stem, and fruit of cucumber (Supplementary Data Fig. S1; Fig. 2) [32, 39]. Spatial positioning analysis further showed that *CsNADP-ME2* transcripts and proteins are highly expressed in exocarp and vasculature in cucumber fruits (Fig. 3).

It has been reported that *cis*-elements such as an ancient G-box in the promoter, or those in untranslated regions (UTRs) of decarboxylase genes, *NADP-ME* and *NAD-ME*, are sufficient for bundle sheath (BS)-specific expression in either C_4_ plants such as maize or mid-vein expression in C_3_ species such as *Arabidopsis*. These observations suggested a possible transcriptional and/or post-transcriptional regulation of *NADP-ME* and *NAD-ME* [21, 26, 40]. Similarly, prediction of the *NADP-MDH* promoter region in sorghum (*Sorghum bicolor*) leaves indicated that there is transcriptional regulation related to mesophyll cell-specific expression of *NADP-MDH* [41]. Upstream open reading frames (uORFs) act as translational or post-transcriptional control elements to regulate gene expression by modulating main open reading frame (mORF) translation in the plant [42]. By affecting translation initiation of the mORF and/or inducing nonsense-mediated decay in a *cis*-acting manner [43], uORF-based gene regulation has been demonstrated to play an important role in plant metabolism [*43*–*46*], such as conditioning the metabolism of spermidine [44], phosphocholine [45], phosphate [43], and ascorbate [46]. In *Arabidopsis*, a mitochondrial dicarboxylate carrier (DIC1) is predicted to belong to the plant conserved peptide uORF family, and functions as a malate/oxaloacetate shuttle to provide other cellular compartments with reducing equivalents [47, 48]. In cucumber, we previously revealed that an uORF in the 5′-UTR of *CsHT1* mRNA, which encodes a pollen-specific hexose transporter, may modulate the translation of *CsHT1* [49]. In the present study, we found that there was indeed a certain number of uORFs in the predicted transcripts of those family genes, e.g. one uORF in *CsNADP-ME2*, *CsNAD-ME2*, and *CsPEPCK1*, two uORFs in *CsNADP-ME1*, and six uORFs in *CsPPDK* (Supplementary Data Table S1). uORFs are selected during evolution and their regulatory activities are modulated by varying environments and tissues to accelerate local adaptation, domestication, and improvement in plants [42]. In this study, the trend of both spatiotemporal expression and enzyme activity seemed to be inconsistent between cucumber fruits and leaves (Fig. 2; Supplementary Data Fig. S3), indicating that these enzymes in cucumber, especially decarboxylases, are most likely under some certain degree of post-transcriptional or translational regulation caused by uORF variations.

At the post-translational level, modifications of maize ZmC_4_-*NADP-ME* at Ser419 (S419) altered enzymatic activity during the day, which might coordinate the carbon concentration mechanism with the rate of CO_2_ fixation [50]. Additionally, the phosphorylation state of PEPCK in many plants modulates its enzyme activities [*51*–*54*]. Likewise, light intensity, the light/dark transition, and high temperature can regulate pyruvate phosphate dikinase (PPDK) activity by modulating reversible phosphorylation [*55*–*57*]. However, whether there is most likely extensive (post-)transcriptional and/or (post-)translational regulation of these enzymes, including three decarboxylases and PPDK in cucumber fruits and leaves, remains to be further explored.

### *CsNADP-ME2* may play roles in balancing carbon and nitrogen metabolism via continuous bottom-up feedback regulation of glycolysis in cucumber fruits

Plant NADP-ME is a key enzyme in malate metabolism [11]. Malate partitioning is closely related to starch accumulation. In many cases, malate and starch levels have a remarkable negative association [13]. By manipulating the genes of malate metabolism enzymes, for example, transgenic tomato green fruits with knockdown of mitochondrial MDH with RNA interference technology [13] and transgenic potato plants with constitutive upregulation of a physiologically active engineered PEPC [58] both saw a considerable rise in the levels of malate but a remarkable drop in soluble sugar and starch contents. Moreover, the downregulation of either plastidic NADP-ME or cytosolic PEPCK resulted in a decrease of starch content in tomato fruit at breaker stage [59]. In the present study, the malate content significantly decreased in *CsNADP-ME2*-OE fruits, especially in the exocarp (Fig. 6A), while the starch content dramatically increased (Fig. 4I). By contrast, there was little change in starch following the increase of malate content in *CsNADP-ME2*-RNAi fruit (Figs 4I and6A). Accordingly, similar to what happened in tomato fruit [59], we postulate that the lower variation in starch metabolism caused by downregulation of *CsNADP-ME2* in cucumber fruit may not be related to altered malate levels per se but potentially to altered redox status. However, future research should be conducted to corroborate this hypothesis.

In plants, the metabolism of carbon and nitrogen are intimately intertwined [60]. In C_4_ plants, there is ample evidence of an interaction between C_4_ pathways and the levels of nitrogen [61, 62]. The mid-vein concentration of sugar and its derivatives was mainly impacted in *Arabidopsis* by lowering cytosolic NADP-ME activity, with severe impacts on glycolytic intermediates, including glucose-6-phosphate and fructose-6-phosphate, whereas abolishing the activity of NAD-ME, another biochemical subtype of decarboxylase, primarily influenced the levels of glucosamine and amino acids in the mid-veins [21]. According to metabolic profiling analysis of tomato fruits at the breaker stage, inhibiting plastidic NADP-ME would increase some amino acids in the pericarp tissues, such as aspartate, serine, and valine, as well as sugars and their derivatives, like *myo*-inositol and fructose [59]. In addition, amino acids were observed to be accumulated in mutant *NADP-ME1* loss-of-function embryos developed from aged seeds in *Arabidopsis* [20]. Hence, the effect of malate decarboxylase on carbon and nitrogen metabolism probably depends on the biological subtype of decarboxylase, the plant tissue, and the developmental stage.

In cucumber, manipulating the expression of *CsNADP-ME2* had significant effects on both sugar levels (Fig. 4) and amino acid contents in fruits (Fig. 6), in general manifesting opposite trends between the metabolism of nitrogen and C, in either OE plants or RNAi lines (Fig. 6). These data illustrate that cucumber *CsNADP-ME2* is likely involved in the balance between the metabolism of nitrogen and carbon by modulating the direction of carbon flow in fruits, since malate provides a continuous bottom-up feedback regulation of PK- and PFK-mediated glycolysis, i.e. positive feedback in *CsNADP-ME2*-OE plants and negative feedback in *CsNADP-ME2*-RNAi lines (Fig. 6). Therefore, based on gene expression analysis, tissue-specific localization and the metabolic and molecular genetic tests presented in this report, we propose a putative model of *CsNADP-ME2* function in cucumber fruit (Fig. 7). We also hypothesize the potential role of *CsNADP-ME2* in the balance between the metabolism of nitrogen and carbon in cucumber fruit, which involves coordination of several physiological processes including glycolysis, amino acid metabolism, CO_2_ fixation, the TCA cycle, and photorespiration (Figs 6 and7).

Taken together, our data confirmed that CsNADP-ME2 catalyzed malate decarboxylation to generate pyruvate and CO_2_ in the exocarp, main vascular bundle, and placental tissues of cucumber fruit. In addition, the results provide new insight into the role of a malate decarboxylase CsNADP-ME2 in regulating the carbon flux into sugars/starch and amino acids in fruits of cucumber. Complex processes may be implicated in reactions of higher plants to the nitrogen–carbon balance, but the knowledge gathered from this work offers novel insights into the molecular genetics and metabolic alterations in nitrogen–carbon balance in various plants.

## Materials and methods

### Plant materials, growth conditions, and treatments

A phytotron under conditions that included a 10-h photoperiod and a 25°C/18°C (day/night) temperature cycle was used to pre-culture cucumber (*C. sativus* cv. ‘Xintaimici’) seedlings. Subsequently, 500 μmol m^−2^ s^−1^ of photon flux density was used as the light intensity. Three replicates of a randomized design were used to position the seedlings in a solar greenhouse. Greenhouse management followed the same schedule as that used by local growers. Fruits from cucumbers were harvested at 2 days before anthesis and 0, 3, 6, and 9 DAA. Cucumber leaves were collected 1 and 9 days after unfolding (DAU).

### Extracting RNA and analysis with real-time quantitative PCR

The Quick RNA isolation kit standard protocol (Huayueyang Biotechnology Co. Ltd, Beijing, China) was followed for extracting RNA. DNase was subsequently employed to eliminate any remaining residue DNA from the isolated RNA. PowerScript™ Reverse Transcriptase (Tiangen Biotechnology Co. Ltd, Beijing, China) was employed to create cDNA. The RT–PCR system (20 μL) contained 1 μL of dNTP mixture (10 mM), 1 μL of oligo dT primer (2.5 μM), 2 μg of total RNA, and RNase-free ddH_2_O to a volume of 10 μL. After denaturation at 65°C for 5 min, 4 μL of 5× reverse transcriptase buffer, 0.5 μL of RNase inhibitor (40 U/μL), 0.5 μL of reverse transcriptase, and 5 μL of RNase-Free ddH_2_O were added in sequence. The reverse transcription program was as follows: 42°C for 15 min, followed by 95°C for 5 min.

RT–qPCR was conducted in an optical 96-well plate with a 7500 Real-Time PCR System from Applied Biosystems (http://www.appliedbiosystems.com/) using SYBR^®^ Green to track the production of double-stranded DNA. Primers (Supplementary Data Table S2) that exhibited similar amplification efficiency were selected. Ten microliters of 2× SYBR^®^ Green Master Mix reagent (TaKaRa, Beijing, China), 1 μL cDNA, and 125 nM of each gene-specific primer were used in the reactions, which had a final volume of 20 μL. Each set of cDNA samples and primer pairs underwent three biological and three technical repetitions. The data from the various tissues were then normalized depending on the expression level of the constitutive *β-tubulin* (*TUB*) genes.

### Phylogenetic analysis

NADP-ME protein sequences used for phylogenetic trees were downloaded from the TAIR database (https://www.arabidopsis.org/) and the National Center for Biotechnology Information (NCBI, http://www.ncbi.nlm.nih.gov) (Supplementary Data Table S3). By using the neighbor-joining method with 1000 replicates in MEGA X [63], the full-length sequences of NADP-ME proteins were compared to construct a phylogenetic tree. The photosynthetic isoforms were named C_4(1)_-NADP-ME and CAM_1_-NADP-ME, the plastidic non-photosynthetic NADP-ME isoforms were named C_4(2)_-NADP-ME and C_3(2)_-NADP-ME, while the non-photosynthetic cytosolic isoforms were named C_4(3)_-NADP-ME, CAM_2_-NADP-ME, and C_3(1)_-NADP-ME [15].

### *CsNADP-ME* gene cloning and cucumber transformation

A complete *CsNADP-ME2* coding sequence was retrieved from the Cucurbit Genomics Database (Cucumber Chinese Long Genome v2, http://cucurbitgenomics.org/organism/2), followed by the use of specific primers for the amplification of the complementary DNA (cDNA) from the cucumber fruit (Supplementary Data Table S2), and subsequent cloning into pGEM-T Easy (TaKaRa, Beijing, China) vector. uORF prediction was performed using the sequence structure (ATG-3n-TAG|TAA|TGA) from 5′-UTRs of syntenic genes, which had lengths ranging from 30 to 1000 bases [49]. The uORF analysis of other genes related to malate metabolism was similar.

For overexpression, the *CsNADP-ME2* ORF was inserted into the expression vector pBI121. Fragments of *CsNADP-ME2* were amplified with specific primers (Supplementary Data Table S2). Using pFGC-1008, an RNA interference vector was created by conventional techniques. Using the cotyledon transformation mediated by *Agrobacterium tumefaciens* (LBA4404), these recombined vectors were individually transformed into the cucumber cultivar ‘Xintaimici’ [49]. The selection medium was used to screen transgenic lines, and their DNA and RNA contents were assessed via PCR and RT–qPCR techniques. *T*_2_ transgenic lines were used for further study.

### Feeding of ^14^C, imaging, and thin-layer chromatography

Radiolabeling with ^14^C and autoradiography studies were carried out according to previously reported methods [21, 24, 33]. The roots of hydroponically grown cucumbers were fed with 3.7 × 10^6^ Bq NaH^14^CO_3_ solution for 2 h under light and left for 1 h before sampling. Fruit for autoradiography was sectioned before being flash-frozen at −80°C and allowed 3 days of freeze-drying. Once the tissue had dried, it was pressed flat and autoradiographed for 14 days with Kodak BioMax MR-1 film (Rochester, NY, USA).

The [U^14^C] malate feeding experiment was conducted as described by Centeno, *et al*. [13] with minor modifications. A fresh incubation medium (10 mM MES-KOH, pH 6.5) was used to wash the cucumber exocarp, MVB, and placenta tissue three times before being allowed to incubate [eight discs in 5 mL of incubation medium containing [U^14^C] malate (1.4 MBq mmol^−1^)] in malate to a 10 mM final concentration. Following a 2-h incubation period, samples were washed three times again in an unlabeled incubation medium before being frozen in liquid nitrogen until subsequent examination. A 100-mL sealed flask was used for each incubation, which was conducted at 25°C under light with a 150-rpm shaker.

Extraction of soluble products was conducted as described previously [21]. The samples were successively isolated in 80% ethanol (v/v) at 70°C for half an hour, 100% (v/v) acetone at 40°C for half an hour, and 80% (v/v) ethanol at 70°C for half an hour in sequence. A vacuum oven was used to dry down the supernatant, followed by rehydration in ddH_2_O. Thereafter, using 20-cm long silica plates (250 μm, Silica Gel 60 A; Merck, Germany), TLC was used to evaluate these samples. Organic acids were separated in *tert*-butanol:acetone:ammonia:water (7:5:3:2 by volume) three times as described by Brown *et al*. [21] . Carbohydrates were separated in acetic acid:chloroform:water (7:6:1 by volume) three times [64]. The plate with radiolabeled spots was exposed to autoradiography film (Kodak Biome MR film, Rochester, NY, USA, https://www.sigmaaldrich.com/catalog/product/sigma/z350400?lang=zh&region=CN), and after scraping away the spots from the plates, Ecoscint scintillation solution (National Diagnostics) was used to count them. To identify the labeled compounds, parallel plates containing standards of organic acids, amino acids, and carbohydrates were run. Amino acids, carbohydrates, and organic acids were observed with bromocresol green (NaOH adjusted), 50% (v/v) sulfuric acid, and ninhydrin solution, respectively [21]. The percentages of radiolabeled amino acids, sugars, and organic acids were expressed as the proportion of total material.

### Enzymatic assays

The tissue of frozen leaves or fruits was extensively crushed under liquid nitrogen. For NADP-ME extraction, the cucumber sample was suspended thoroughly in an extraction buffer [10 mM 2-mercaptoethanol, 10% (v/v) glycerol, 2 mM EDTA, 5 mM MgCl_2_, and 100 mM Tris–HCl (pH 8.0)] [59]. The supernatants were obtained after spinning at 13 000 g for 10 min to determine NADP-ME activity in the reaction mix containing 10 mM malate, 0.5 nM NADP, 10 mM MgCl_2_, and 50 mM Tris–HCl. The reaction was initiated by adding malate [22]. Using a spectrophotometer (Unico UV-2802PC, USA), activity was determined by tracking the rise in NADPH absorbance at 340 nm.

Extraction and enzyme activity of PEPC, NAD-ME, and PPDK were examined as indicated [65]. Monitoring NADH oxidation at 340 nm at room temperature allowed the measurement of PEPCK activity in the carboxylation direction [39]. NADP-malate dehydrogenase (NADP-MDH) was extracted and the activity was measured as described previously [66]. Spectrophotometric analysis was used to assess the activities of PFK and PK as indicated in Liu *et al*. [67]. The treated samples were incubated at 25°C for 10 min and the increase in absorbance at 340 nm was recorded for 10 min.

### *In situ* hybridization, western blot, and immunolocalization localization

*In situ* hybridization (ISH) and immunolocalization localization were conducted as outlined by Sui *et al*. [30] with minor changes. To mount cross-sections on ProbeOn Plus Slides (Thermo Fisher Scientific, Runcorn, UK) for ISH, they were cut to a thickness of 10 μm. The DIG RNA labeling kit (Roche, USA) was used to create sense and antisense riboprobes for ISH by *in vitro* transcription using the SP6 or T7 promoter from PCR products. Supplementary Data Table S2 gives a summary of the primer sequences. A dot-blot test was used to determine the AgMaT1 probe’s selectivity. ISH was conducted as outlined by Jackson [68]. Images were captured with an Olympus BX53 microscope.

Rabbits were immunized by MBL Beijing Biotech Co. Ltd (Beijing, China) using a specific peptide fragment (MESTLKEIGDGGSVLDLD) produced from the CsNADP-ME2 protein sequence. Separation of samples was done by SDS–PAGE before blotting them onto nitrocellulose and then treating them with an anti-CsNADP-ME2 antibody for the western blot assay. After washing several times, the membranes were incubated with goat anti-rabbit IgG antibody–horseradish peroxidase conjugate (Abmart, Shanghai, China). Proteins were visualized using electrochemiluminescence (ECL) reagents (Millipore, USA). To conduct immunolocalization, we blocked the cross-sections and allowed them to incubate in the primary CsNADP-ME2 antibody (diluted 1:500), followed by incubation with a 1:200 dilution of secondary antibody (goat anti-rabbit AP or IgG–FITC labeling). An Olympus BX53 microscope (AP labeling) or Olympus Fluoview FV1000 confocal laser scanning microscope (FITC labeling) was employed to capture the images with an excitation wavelength of 488 nm, and observations of chloroplast fluorescence were made at a 546 nm wavelength.

### Gas exchange

Gas exchange was performed according to a previous study [30]. With the aid of a plexiglass fruit chamber that was equipped with a fan and a CO_2_ gas sensor system (GXH-3052 L, Jun-Fang-Li-Hua Technology Research Institute, Beijing, China), the rates of CO_2_ evolution for whole fruits, peels, and slices without peels were assessed in both the dark and light condition. To avoid CO_2_ exchange at the tape–tissue interface, peels and slices were applied to the wet plastic tape. Wound respiration of slices caused by cutting was determined and corrected [30]. Net photosynthesis rate, and proportion of recaptured CO_2_ were analyzed according to Sui *et al*. [30].

### Metabolite profiling

Samples were ground to a fine powder, and 50 mg of ground tissue from each independent pool was used for further extraction. To conduct chromatography–mass spectrometry (GC–MS), metabolite extraction, derivatization, standard addition, and sample injection were carried out as indicated by Schauer *et al*. [69]. TagFinder was utilized to assess chromatograms and mass spectra [70].

### Statistical analysis

Analysis of variance (ANOVA) was conducted to statistically examine experimental data using SPSS software version 14.0. (SPSS Inc., Chicago, IL, USA). Tukey's tests were used to detect significant differences.

## Acknowledgements

This work was supported by the National Natural Science Foundation of China (32272695 and 31972398 to X.S.), the National Key Research and Development Program of China (2019YFD1000300), the National Natural Science Foundation of China (31960591 to N.S.), the Max-Planck Society and European Union’s Horizon 2020 research and innovation programme, project PlantaSYST (SGA-CSA No. 664621 and No. 739582 under FPA No. 664620), the China Agriculture Research System of MOF and MARA (CARS-23), and the 111 Project of Ministry of Education of P.R.C. (B17043).

## Author contributions

X.S. and N.S. conceived the project and designed the experiments; N.S., Y.Z., Y.G., W.Z., and J.N. performed the experiments; Y.Z., W.Z., and A.R.F. provided technical assistance to X.S., N.S., and Y.G.; N.S., Y.Z., Y.G., and X.S. analyzed the data. N.S., X.S., and Y.Z. wrote the article, and A.R.F revised the article. All authors read and approved the final draft of the manuscript.

## Data availability

The accession numbers of all genes used in this paper were obtained from the Cucurbit Genomics Database (Cucumber Chinese Long Genome v2 http://cucurbitgenomics.org/organism/2) and are listed in Supplementary Data Table S3.

## Conflict of interest

The authors declare that they have no conflicts of interest.

## Supplementary data

Supplementary data is available at *Horticulture Research* online.

## Supplementary Material

Web_Material_uhad216
