# Supplementary material for: Cucumber malate decarboxylase, CsNADP-ME2, functions in the balance of carbon and amino acid metabolism in fruit
Source: Hortic Res. 2023 Oct 25;10(12):uhad216. doi: 10.1093/hr/uhad216 (PMC10699846; doi:10.1093/hr/uhad216)
Supplement: Web_Material_uhad216 [file web_material_uhad216.pdf]

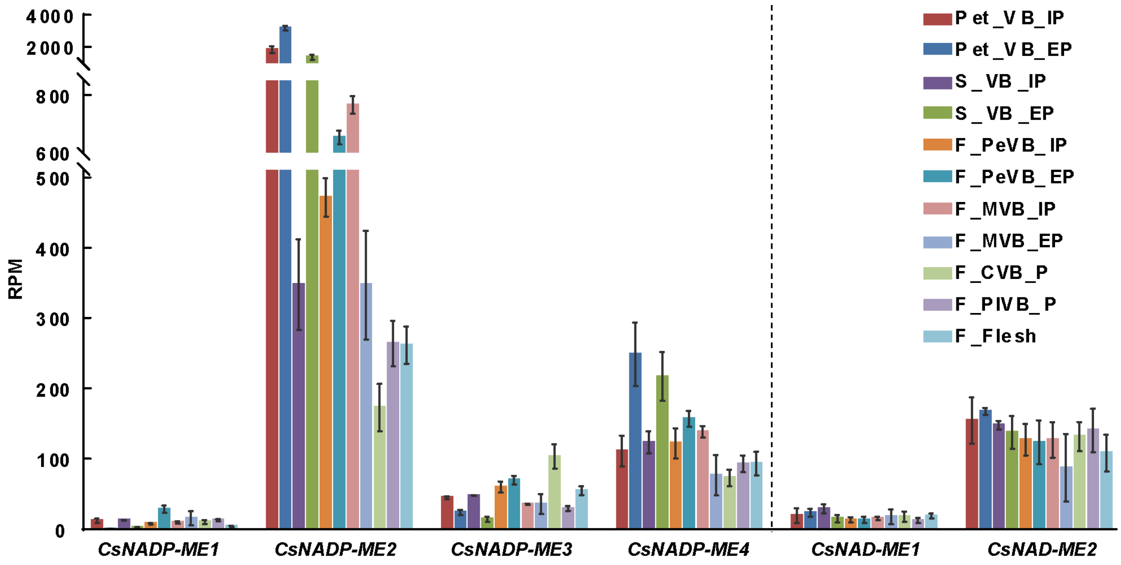

Supplementary Fig. S1 Expression profiles of C_4 acid decarboxylases including NADP-ME and NAD-ME encoding genes by laser capture microdissection (LCM)-derived RNA-seq analysis in phloem systems of cucumber shoot and ovary/fruit during early development. For detailed experimental methods, please refer to Sui *et al.* (2018; 2021). Expression profiles of PEPCK encoding genes in different phloem tissues of cucumber, also please refer to Supplementary Fig. S6 in Sui *et al.* (2021). Mean values ± SE of three independent biological replicates are shown. Abbreviations: NADP-ME, NADP-malic enzyme; NAD-ME, NAD-malic enzyme; PEPCK, phosphoenolpyruvate carboxykinase; Pet, petiole; S, stem; F, fruit; VB, vascular bundle; PeVB, MVB, CVB, PIVB, peripheral, main, carpel and placental vascular bundle; IP, internal phloem; EP, external phloem; P, phloem; RPM, Reads per million.

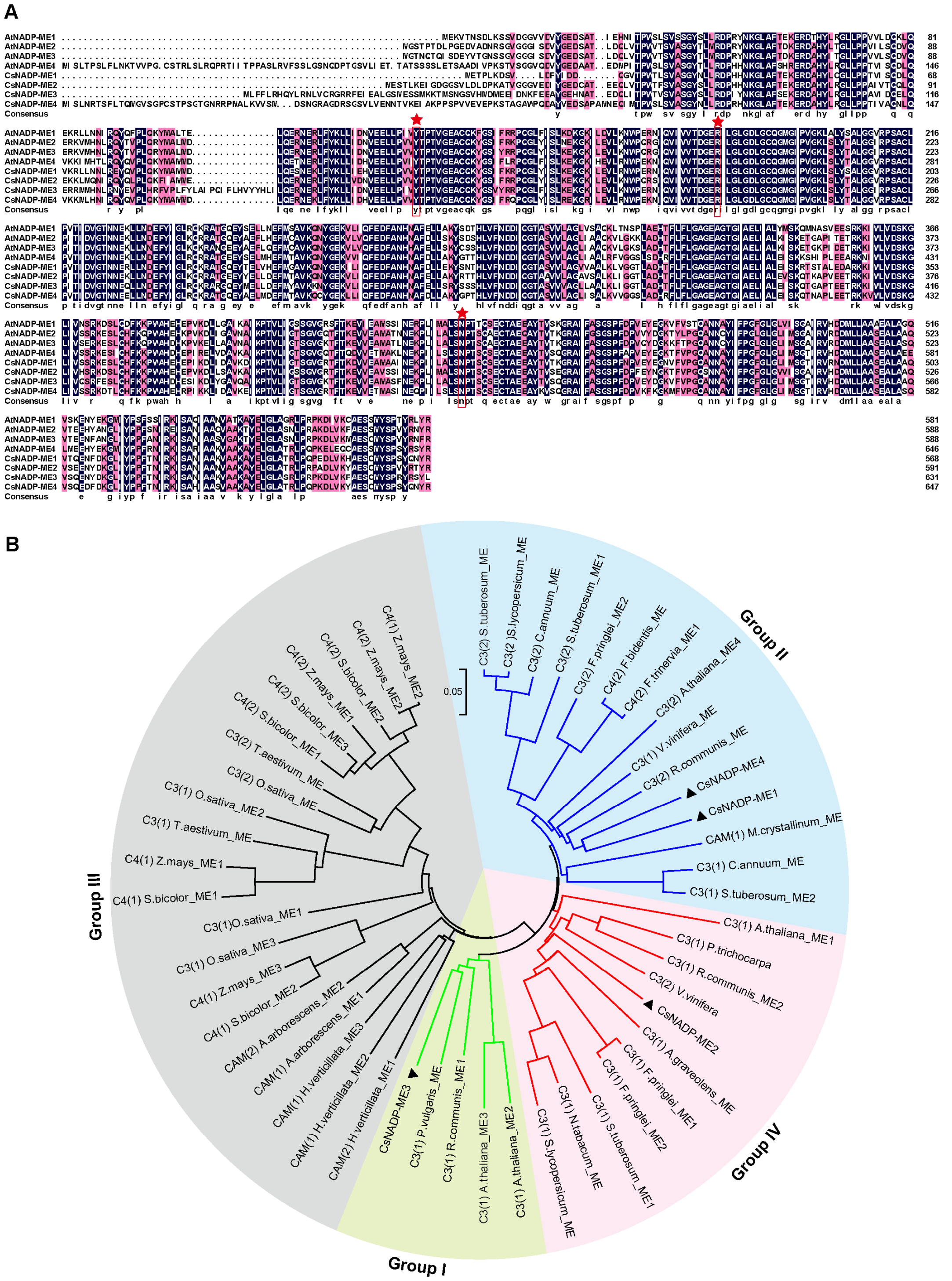

Supplementary Fig. S2. Alignment (A) and phylogenetic tree analysis (B) of NADP-ME proteins. (A) Four candidate NADP-ME proteins named from CsNADP-ME1 to CsNADP-ME4 in cucumber and four homologous Arabidopsis AtNADP-ME proteins were identified. Dark blue boxes indicate identical amino acid residues. Red boxes represent amino acid residues with an identity of more than 75%. The sequence alignment was performed using the DNAMAN software package. Strong conservation of active site residues (red stars) involved in binding

malate/pyruvate was observed in *Arabidopsis* and cucumber orthologs. (B) Predicted amino acid sequences were aligned using MEGA X adopting Poisson correction distance by bootstrap method with 1000 replicates, and it was presented as a traditional rectangular tree view. The following sequences, in addition to the four cucumber (*Cucumis sativus*) NADP-MEs, were analyzed from *Aloe arborescens*, common ice plant (*Mesembryanthemum crystallinum*), hydrilla (*Hydrilla verticillate*), Arabidopsis (*Arabidopsis thaliana*), castor bean (*Ricinus communis*), *Apium graveolens*, bean (*Phaseolus vulgaris*), *Flaveria pringlei*, grape (*Vitis vinifera*), *Capsicum annuum*, Black cottonwood (*Populus trichocarpa*), potato (*Solanum tuberosum*), rice (*Oryza sativa*), tobacco (*Nicotiana tabacum*), tomato (*S. lycopersicum*), bread wheat (*Triticum aestivum*), maize (*Zea mays*), sorghum (*Sorghum bicolor*), *F. trinervia*, *F. bidentis*. GenBank accession numbers and sources are listed in Supplementary Table S3. The photosynthetic isoforms were named $C_{4(1)}$-NADP-ME and CAM_1-NADP-ME; the plastidic nonphotosynthetic NADP-ME isoforms as $C_{4(2)}$-NADP-ME and $C_{3(2)}$-NADP-ME, while the nonphotosynthetic cytosolic isoforms as $C_{4(3)}$-NADP-ME, CAM_2-NADP-ME, and $C_{3(1)}$-NADP-ME, respectively The phylogenetic tree can be divided into four groups, including **Group I**: cytosolic dicot NADP-ME group; **Group II**: plastidic dicot NADP-ME group; **Group III**: monocot NADP-MEs; and **Group IV:** a group composed of cytosolic monocot and dicot NADP-MEs.

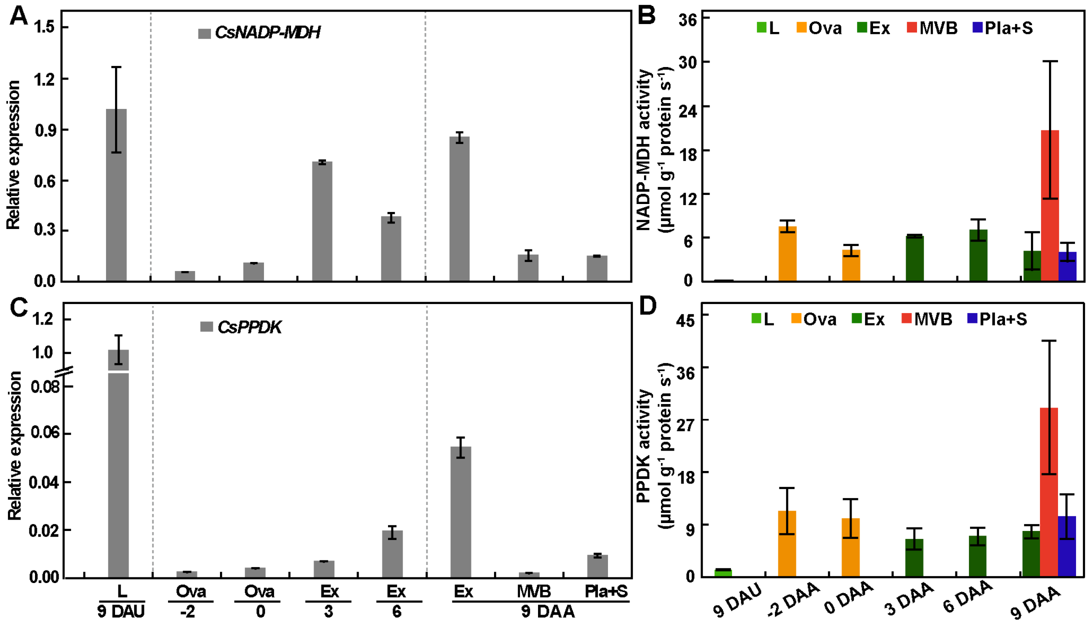

Supplementary Fig. S3 Transcript levels and enzymatic activities derived from *CsNADP-MDH* and *CsPPDK* were analyzed. (A, C) Real-time quantitative PCR (RT-qPCR) of *CsNADP-MDH* (A) and *CsPPDK* (C) was conducted on cDNA derived from cucumber mature leaves and different fruit tissues. Specific primers used for each gene are listed in Supplementary Table S2, and PCR was performed as a duplex reaction with primers for *β-tubulin*. (B, D) Enzymatic activities of NADP-MDH (B) and PPDK (D) from cucumber mature leaves and different fruit tissues. The enzymatic activities were calculated per protein concentration. Abbreviations: *NADP-MDH*, *NADP-dependent malate dehydrogenase* (Gene ID, Csa5G622460); *PPDK*, *pyruvate orthophosphate dikinase* (Gene ID, Csa3G150740); DAA, days after anthesis; DAU, days after unfolding (of leaves); Ex, exocarp; L, leaf; MVB, main vascular bundle; Ova, ovary; Pla, placenta; S, seeds.

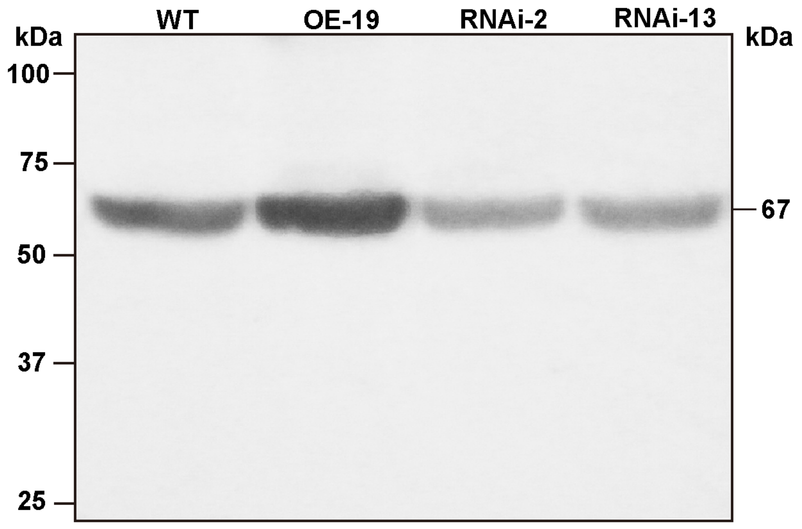

Supplementary Fig. S4 Specificity analysis of anti-CsNADP-ME2 antibody. Western blot of CsNADP-ME2 to test the quality of the anti-CsNADP-ME2 antiserum in the sample extracted from fruit exocarp tissues of WT, OE-19, RNAi-2, and RNAi-13. Compared with WT, a specific 67-kDa band was significantly deepened in the OE-19 exocarp, while the corresponding bands were significantly weakened in RNAi-2 and RNAi-13 exocarp tissues.

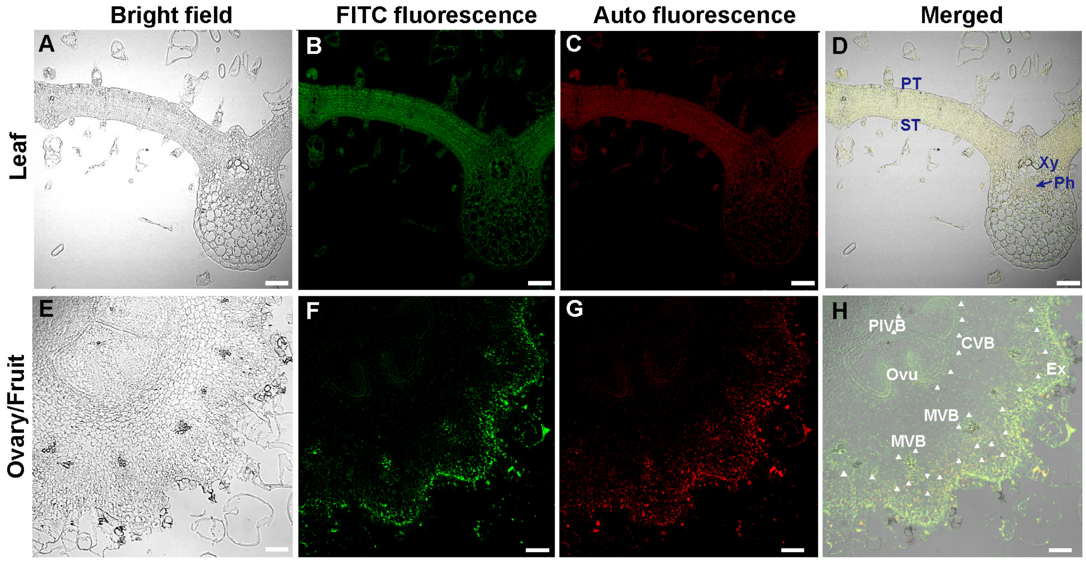

Supplementary Fig. S5 CsNADP-ME2 localization via fluorescence immunohistochemistry in cucumber fruits and leaves. The cross-sections of leaves (0-1 DAU, days after unfolding) (A-D) and young ovaries/fruits (-2-0 DAA, days after anthesis) (E-H), respectively. The secondary antibody was fluorescein isothiocyanate (FITC) labeled anti-rabbit IgG. Green color indicates fluorescence from FITC, and red indicates auto fluorescence from chlorophyll in the chloroplasts. White triangles indicate signals. Scale bars: 50 μm in (A-D), and 100 μm in (E-H). Abbreviations: Ex, exocarp; Ovu, ovule; Ph, phloem; PT, palisade tissue; ST, spongy tissue. Xy, xylem; MVB, CVB, and PlVB, main, carpel and placental vascular bundle.

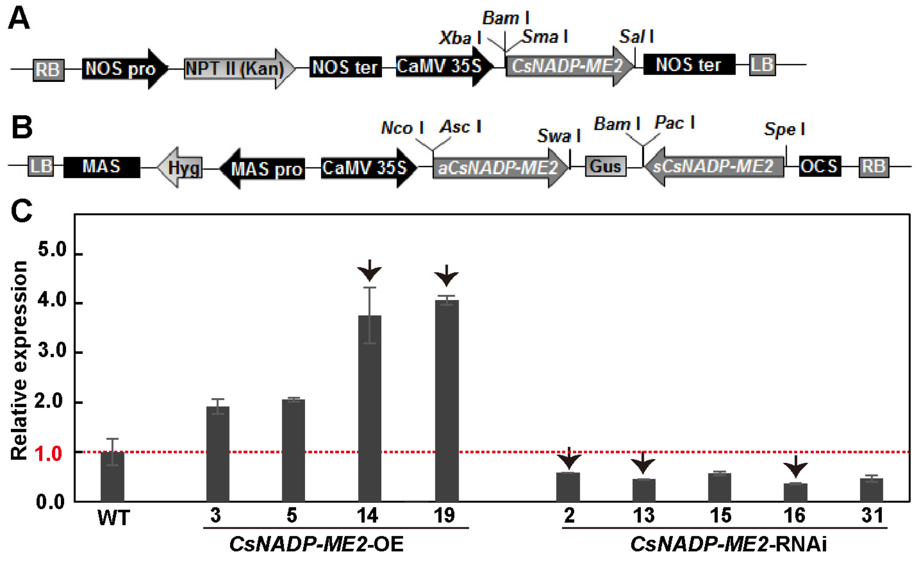

Supplementary Fig. S6 Construction of transgenic vectors of cucumber *CsNADP-ME2* and selection of cucumber transgenic lines. (A, B) Schematic structure of the overexpressing (OE) (A) and RNA interference (RNAi) expression vectors (B) of *CsNADP-ME2*. (C) Transcript levels of *CsNADP-ME2* in the exocarp tissues of OE and RNAi T_0 lines, and the arrows show the selected lines for further analysis.

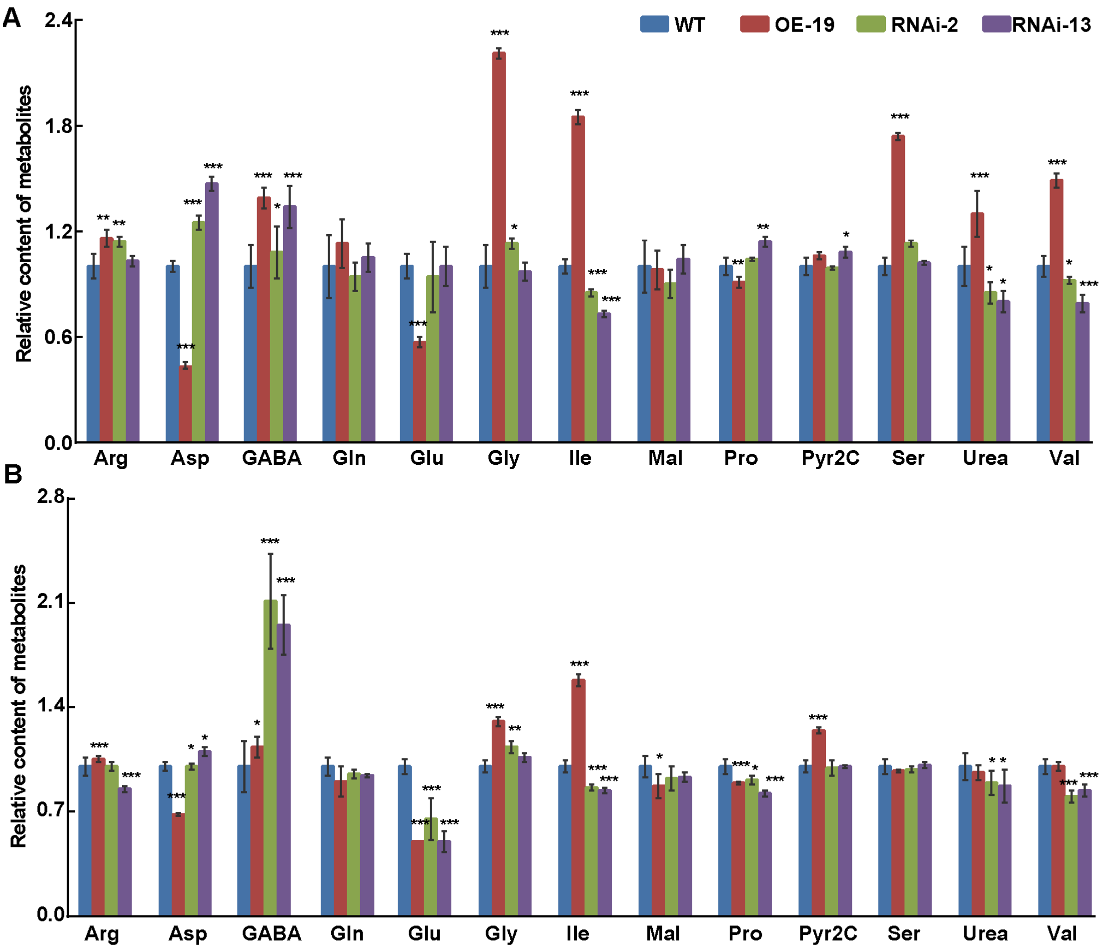

Supplementary Fig. S7 Analysis of altered primary metabolism in fruit MVB (A) and placenta tissues (B) of *CsNADP-ME2* transgenic plants. Three transgenic lines (OE-19, RNAi-2 and RNAi-13) were selected for study and WT plants as control. Compared to WT, in *CsNADP-ME2*-RNAi lines, the levels of Asp and GABA were significantly up-regulated in both MVB and placenta tissues, while the Ile, the Val and Urea levels were dramatically down-regulated. By contrast, in the MVB and placenta tissues of *CsNADP-ME2*-OE plants, Asp and Glu contents were significantly decreased, whereas the contents of Arg, GABA, Gly, Ile and Pyr2c were increased markedly, specially, elevated content of Ser, Val and Urea were found in the fruit MVB. These results showed that the primary metabolic network mediated by *CsNADP-ME2* in cucumber fruit can be very complex, and the changes and regulation of metabolite levels in specific pathways might depend on different tissues or spatial sites. Metabolite data are normalized to mean values of the wild type. Mean values ± SE are given (n = 5). The means followed by different stars indicate statistically significant differences compared to WT according to Tukey's test (*$P < 0.05$, ** $P < 0.01$, ***$P < 0.001$). Abbreviations: Arg, arginine; Asp, aspartate; GABA, γ-aminobutyric acid; Gln, glutamine; Glu, glutamate; Gly, glycine; Ile, isoleucine; Mal, malate; MVB, main vascular bundle; Pro, proline; Pyr2C, 1-pyrroline-2-carboxylate; Ser, serine; Val, valine.

Supplementary Table S1. The upstream open reading frames (uORFs) analysis in the 5' untranslated region (5' UTR) sequence of malate metabolism-related genes.

Sequence structure of (ATG-3n-TAG|TAA|TGA) were used to predicted uORFs in the 5’ UTR of genes as mentioned above. uORF, upstream open reading frames, were highlighted in blue; The uORF ATG was highlight with a single underline, and uORF stop codon was indicated by a double underscore. 5’ UTR and uORF were not found in *CsNAD-ME1* gene sequence, and indicated by dash line.

Abbreviations: mORF, major open reading frame, was highlighted in red; 5’ UTR, 5’ untranslated region.

Gene name	Sequence of 5’ UTR and mORF	Number of uORF
CsNADP-ME1	TTTGATCTGTTGAGGGAGAGAGAGTTGGGAGGAAGTTGAAAGGTGGAAGAAAGA**ATGGAATCCTACATACCCCATTAA**AAGGGCTTTAATTTGTTGTGTTG<br>TTCCTTAATAATCTCTCATTTGTTTCTCTTTGTGTTTTCTTCCCCCAC**ATGCATCCATCCAACCCAAAGACTATTTCTAGCTCTTTCCCTTTCTCCTCCACCAC**<br>**ATCCCTCACCAATTCATAA**ATCTATCTTCTGGCCGGTTCCTCTTGTTTAATTTTATTTTAACACAACAACAAACACCCACATTTCTTCTACTCTTATAGCTGTA<br>TCTCTCACAATCCTTCAACTTCAACCTCTCTCTTCCATCTGTTAGATCAGAGACCACCACGTACGTTTTCATG…...	2
CsNADP-ME2	TATCTTTCGCCAAATTTGACTTTGCCTTTTCTCCTCGTCAATGGTGGCAAGTGGGTTTGGTTATTTA**ATGGACGCCCAAGTCTTCCCAATTCGTATTGTGTTT**<br>**ATTTTTTGA**GGAAGATTTGTTTTGCCACTCTTCTTTTTGTGGGTTGCTTCAATTTTGAGTTTAGTGATCTGGGATG……	1
CsNADP-ME3	TCCTTCTTCTTTTCTTTTCATCCTAATTTCTATTTCCATTTTCACAATTATG……	0
CsNADP-ME4	CCTCTTTTCCCCTCCTCTTTCACCACTATCACTTTTCACCATG……	0
CsPEPCK1	AGAGCCCTTCCGTTCTTCCACTTCCTCCAAGCCATTAACTCCAAACAATAAAACTTATAAATACCCATTACTCACCCATTTTCCAATTCATTCTTCTCTCATATT<br>TCAACTTCTCTTCCTCTTATCCATCACAAATCTCCGGTACCCACCTACCCTTTCTCTCT**ATGTTTTCGTTTTTTTAA**TGCGATTTTCAGTTCTTTCATTTTTTTCTT<br>CTGTTAACTAAGTGTAATTGACGAATTCAATCTTCGACTGTGTTGCAGAGGATACAAAAAATG…...	1
CsPEPCK2	ATTCATCAAAACTCAACCCCCCTCTCTCTCGTTCTCTCTGCATTCACACTTTTGATTTCATTCATCTCATAACCGTTCTTTGCCAAACTTCTGTACGTCTACATT<br>TCTCCTATTCCCTCTCTTTTTTTTATTTCTAAACGATTACTCCAACTCTTTAACATGACAATTAGACAATTGAATTTTTATTTCCATCTGTTTCTTCTTTTTATAGG<br>ATTGACATG……	0
CsNAD-ME1	—	—
CsNAD-ME2	AACCTTT**ATGTTTCAACTGACAAAGCCTCTGAAACCTTAA**CCTTAACTAGCATTGCTTCGAATTACAGCTGATCATTTCTTTTTAATCTTTTTGGAAGATAGG<br>TTGGACACTTGAAGTAGTAGAGCATTGGATTTGCGGCACTGTTGCATTTGGTTTACTGGTAGCCAGATTGGATTTGGTTTCTGCTTTCTTGATTTTTCCCTAG<br>ATCCAGCTTTATG……	1

CsNADP-MDH	CTTTCTTTCTTTCTCCATTTTTCTGCTCAATCTCTATCTCATCTCTCCCGCTCTCTCCCCACCTCCATAGCCAGAAACCTTTACTCCTTCTTCTTCTTCTTTTTC<br>TTCTTCTTCTTCTTCTTATTCTTCTTCTTCTCTCTTCTTCCTTCATTCAATCCCTCTCAATAACATCTCCTTTCCCTTCTTTAAATTCGCGATTTCTCCATTTCTG<br>TTCTCCCTCTGCTGCAATG……	0
CsPPDK	AAAAAAAGCTAACTGTCATTTTCCCCCCTTTGTATTACAGAAACTGTGATCCTCAGCTTCACGAGATTCACACATTAATTTTTTGAAGTTTCTACAACATTGTA<br>TTTTGAAGGTTGCAGCATCTTGTTCCTGTTCTTCTTCTCTTTGTTTTTGGAAGTTCCAAGAGTTTTCTATAGTGAATACTTGGTTCTATTGCTTTAGAGTCTATT<br>ATTTTAAATTAAGATTGACTCTTCTTTGCTGATGCGTCTTATGAATTTGATAGCCCACC**ATGTCCTGTTCTTAA**TGCTCTAGAGATCATTGAGTTTTTAACTTG<br>TGAGTGACCATAATAGGAAATCTGGCTTTCAGGTTTGATTTTTCA**ATGATTTGGGTAACTGCATAA**CTGGTTTGAAAAGTTTCAGTTTGGGGTGTTGTTTTAG<br>TGGGAGTTTGCTAAAAGAAA**ATGTGTGGCACCTACTAA**CTGTCTGTGTTATTTCATTTTCTTGTCTGATACATGGCAGGATTGATTGGAGAGGAAATAATGT<br>CTTCCTTAATGC**ATGGATCACTGCTGCAAACTATAG**CAGATTGTGATCAAGGATTGTTGAGGAGGGGAAAGTACCATTTGGGTCATACCAATCTTCTCAAA<br>GAAAAACATTCATCTTTGAGGGCAAACAGAGGTGGTAAGGGTAAAGGTATTTGCTGCCAAGATTTGCATATCAGTAGTCCAAAGCCAGAGAGACATGAGCC<br>TTCCAACCGTCACGGTTCAAGAGCTGATGCAGTCCTCAGTCCTGTTATACCTACTACCAAAAAGGTTGCTATAT**ATGCTCTTGTGTCTTTTGGTTACTCTTTT**<br>**CATATCGTAA**GTTTTTGGTGGCCATAGTTTATAGCCCCACCACCTAAGTTCTTACCATATTTGTCAATTTGATTATTTTATTGAGTTGTAGTATCCTGTCAGGT<br>TCTTTGGA**ATGCTTAGCTTGTAA**GATGTATATTCTCCTTATGATGTGCAATTTACCTGGTTTTGATTCAATTTCTTGTTTGAACAGAGAGTATTCACTTTTGGA<br>AAAGGAAGGAGTGAGGGTAACAAGAGCATG…...	6

Supplementary Table S2. Primers employed in this study.

**Primers for the amplification of the *CsNADP-ME2* full-length CDS**		
CsNADP-ME2	F	ATGGAGAGTACTTTGAAGGAGATCG
	R	TTATCGGTAGGTTCGGTAGACG
**Primers for the construction of overexpression vector**		
S-CsNADP-ME2	F	TCCCCCGGGATGGAGAGTACTTTGAAGGAGATCG
	R	CGAGCTCTTATCGGTAGGTTCGGTAGACG
**Primers for the construction of RNAi vector**		
S-CsNADP-ME2	F	AGGCGCGCCCTTTGAAGGAGATCGGTGATG
	R	ATTTAAATGAGTAACAATGGCAGGTGGC
A-CsNADP-ME2	F	GACTAGTCTTTGAAGGAGATCGGTGATG
	R	CCTTAATTAAGAGTAACAATGGCAGGTGGC
**Primers for RT-qPCR**		
Q-CsNADP-ME1	F	CACAACAACAAACACCCACATT
	R	GGAGGCAGAAGACCACACAAG
Q-CsNADP-ME2	F	GGCAAGTGGGTTTGGTTATTT
	R	CCTTCAAAGTACTCTCCATCCC
Q-CsNADP-ME3	F	TGAAGAAGACAATGAGCAACGG
	R	GCAACAAGCCACGCAAGTAA
Q-CsNADP-ME4	F	CCCCTCCTCTTTCACCACTATCA
	R	TCCACCAGAACACTCCCGCT
Q-CsPEPCK1	F	CCTCAAGTCCACCCACATCC
	R	AAGCCCCTGTCGACGTTATG
Q-CsPEPCK2	F	CGCCATCAAGTTTGGAACCG
	R	CCGCTCGAGTGTTCTCTGTT
Q-CsNAD-ME1	F	GCTGGACTTTTAGGGGCTGT
	R	GCCATAGTTTTCCTTGCCGC
Q-CsNAD-ME2	F	TTCTTCTTGGTCTGTCAGGAGTT
	R	CAGCGTTCATGGTGGGATTT
Q-CsNADP-MDH	F	ACCACCGTCGCTGCTCTTTC
	R	GCTTTTACTCTTAGGGTCCTCTGTTTTAG
Q-CsPPC1	F	AATACCGTTCCATTGTCTTC
	R	AATCTCGTCTGTGTCCAT
Q-CsPPC2	F	GGTAAGCAAGAAGTTATGATT
	R	GAGATAATATAGCAAGATGAGTAG
Q-CsPPC3	F	TGTGGATGACTTGAGGAA
	R	TTGCCAGAATCAGAATAACC
Q-CscPK2	F	TGAGAAGATATTTTTGGCGCAG
	R	GTGCTTTCAGCTTCAACACATA
Q-CscPK5	F	TTAAGGTGGAGCTTTAGTGGAG
	R	GACTCATTTGTTGCGCTAGTAG
Q-CsPKp1-2	F	CTGCCATAGGATCATATGGACA
	R	GAGCACAAATATGGCGTCTA

Q-CsPKp3	F	ATACAGTTGCTTTACGGACTGA
	R	TGCATGATAAGCAAACATCTCG
Q-CsPFK2	F	AGGAGGGTTTGATCTGAAGAAG
	R	ACGACGTACTTCCTCGAATATC
Q-CsPFK5	F	GATGCATCTGGGAACATTGTAC
	R	TGTTGCAGATTCCTACTGTGAT
Q-CsPFK7	F	CATTCCCTTCTACCGAATCGTA
	R	CCTCTTTCTTCTCCTCCGATAC
Q-CsPPDk	F	TGGGAGGGAAGGGAGCAAAC
	R	CGAACCGAGAGAAGGAGAGG
β-Tubulin	F	GCGTTTGTCTGTTGACTATG
	R	GGATACAAGACGGTTGAGG
**Primers for *in situ* hybridization of *CsNADP-ME2***		
in situ-CsNADP-ME2	F	GATTTAGGTGACACTATAGAATGCTCAATGGTGGCAAGTGGGTT
	R	TGTAATACGACTCACTATAGGGCACAGAACCACCATCACCGA

Supplementary Table S3. List of accession numbers used in this study.

Species	Gene name	Accession number	Species	Gene name	Accession number
Cucumis sativus	*CsNADP-ME1*	Csa7G452890	***Flaveria***	$C_{3\,(2)}$ *F.pringlei* ME2	X78069
	CsNADP-ME2	Csa1G574870	***pringlei***	$C_{3\,(1)}$ *F.pringlei* ME1	AF288920
	CsNADP-ME3	Csa2G373430		$C_{3\,(1)}$ *F.pringlei* ME2	AF288921
	CsNADP-ME4	Csa3G444620	***Vitis vinifera***	$C_{3\,(1)}$ *V.vinifera* ME	NP_001268142.1
	CsPEPCK1	Csa3G893410		$C_{3\,(2)}$ *V.vinifera* ME	NP_001268030.1
	CsPEPCK2	Csa6G497180	***Zea mays***	$C_{4\,(2)}$ *Z.mays* ME1	NP_001105313.1
	CsNAD-ME1	Csa3G127830		$C_{4\,(2)}$ *Z.mays* ME2	XP_008648302.2
	CsNAD-ME2	Csa6G073990		$C_{4\,(1)}$ *Z.mays* ME1	NP_001150965.1
	CsPPC1	Csa4G627210		$C_{4\,(1)}$ *Z.mays* ME2	XP_035815736.1
	CsPPC2	Csa7G048110		$C_{4\,(1)}$ *Z.mays* ME3	NP_001105292.1
	CsPPC3	Csa5G577360	***Capsicum***	$C_{3\,(2)}$ *C.annuum* ME	XP_016550448.1
	CscPK2	Csa4G268000	***annuum***	$C_{3\,(1)}$ *C.annuum* ME	XP_016547705.1
	CscPK5	Csa6G449830	***Solanum***	$C_{3\,(2)}$ *S.tuberosum* ME1	XP_006343041.1
	CsPKp1-2	Csa4G000560	***tuberosum***	$C_{3\,(2)}$ *S.tuberosum* ME2	XP_006359476.1
	CsPKp3	Csa3G359130		$C_{3\,(1)}$ *S.tuberosum* ME1	XP_006353488.1
	CsPFK2	Csa6G510350		$C_{3\,(1)}$ *S.tuberosum* ME2	XP_006364722.1
	CsPFK5	Csa4G097630	***Oryza sativa***	$C_{3\,(2)}$ *O.sativa* ME	D16499
	CsPFK7	Csa1G575110		$C_{3\,(1)}$ *O.sativa* ME1	XP_015620425.1
	CsNADP-MDH	Csa5G622460		$C_{3\,(1)}$ *O.sativa* ME2	XP_015640686.1
	CsPPDK	Csa3G150740		$C_{3\,(1)}$ *O.sativa* ME3	AY444338
Aloe arborescens	CAM $_{(1)}$ *A.arborescens* ME1	AB016804	***Populus***	$C_{3\,(1)}$ *P. trichocarpa*	XP_002324450.1
			trichocarpa		
	CAM $_{(2)}$ A.arborescens ME2	AB005808	***Sorghum***	$C_{4\,(1)}$ *S.bicolor* ME1	Sb09g005810
Arabidopsis thaliana	$C_{3\,(1)}$ *A.thaliana* ME1	AT2G19900	***bicolor***	$C_{4\,(1)}$ *S.bicolor* ME2	Sb03g034280
	$C_{3\,(1)}$ *A.thaliana* ME2	AT5G11670		$C_{4\,(1)}$ *S.bicolor* ME3	Sb03g033250
	$C_{3\,(1)}$ *A.thaliana* ME3	AT5G25880		$C_{4\,(2)}$ *S.bicolor* ME1	Sb03g003230
	$C_{3\,(2)}$ *A.thaliana* ME4	AT1G79750		$C_{4\,(2)}$ *S.bicolor* ME2	XP_002440946.2
Ricinus communis	$C_{3\,(1)}$ *R.communis* ME1	XP_025012663.1		$C_{4\,(2)}$ *S.bicolor* ME3	Sb03g003220
	$C_{3\,(1)}$ *R.communis* ME2	XP_002514230.1	***Nicotiana***	$C_{3\,(1)}$ *N.tabacum* ME	XP_016454656.1
			tabacum		
	$C_{3\,(2)}$ *R.communis* ME	NP_001310668.1	***Solanum***	$C_{3\,(2)}$ *S.lycopersicum* ME	NP_001234458.2
Apium graveolens	$C_{3\,(1)}$ *A.graveolens* ME	AJ132257	***lycopersicum***	$C_{3\,(1)}$ *S.lycopersicum* ME	NP_001233951.2
Flaveria trinervia	$C_{4\,(2)}$ *F.trinervia* ME1	X57142	***Hydrilla***	CAM $_{(2)}$ *H.verticillata* ME1	AY594687
Flaveria bidentis	$C_{4\,(2)}$ *F.bidentis* ME	AY863144	***verticillata***	CAM $_{(1)}$ *H.verticillata* ME2	AY594688
Mesembryanthemum	CAM $_{(1)}$ *M.crystallinum* ME	X64434		CAM $_{(1)}$ *H.verticillata* ME3	AY594689
crystallinum					
Phaseolus vulgaris	$C_{3\,(1)}$ *P.vulgaris* ME	J03825	***Triticum***	$C_{3\,(2)}$ *T.aestivum* ME	EU170134
			aestivum	$C_{3\,(1)}$ *T.aestivum* ME	EU082065
